# Association of PON2 and Its S311C Substitution with Tumor Progression and Its Significance as an Immune-Associated Prognostic Indicator in Clear Cell Renal Cell Carcinoma

**DOI:** 10.3390/cancers18193086

**Published:** 2026-09-23

**Authors:** Zhiyu Liu, Wei Yun, Ting Hong, Chuan Liu, Guangming He, Shenglin Gao, Xiaokai Shi, Li Zuo

**Affiliations:** Department of Urology & Urology Laboratory, The Third Affiliated Hospital of Nanjing Medical University, 68 Gehu Middle Road, Wujin District, Changzhou 213003, China; liuzhiyu@stu.njmu.edu.cn (Z.L.); yunwei@stu.njmu.edu.cn (W.Y.); hongting@njmu.edu.cn (T.H.); liuchuan@stu.njmu.edu.cn (C.L.); heguangming@stu.njmu.edu.cn (G.H.); gaoshenglin12135@njmu.edu.cn (S.G.)

**Keywords:** clear cell renal cell carcinoma (ccRCC), paraoxonase 2 (PON2), prognostic marker, glycolysis, tumor immune microenvironment

## Abstract

**Background:** Clear cell renal cell carcinoma (ccRCC), the predominant histological subtype of kidney malignancies, is characterized by metabolic dysregulation and therapeutic resistance. Although the intracellular antioxidant paraoxonase 2 (PON2) is frequently dysregulated across multiple cancers, its biological role in ccRCC—particularly the contribution of its enzymatic activity and interplay with the tumor immune microenvironment—remains poorly defined. **Methods:** Multi-cohort analysis integrating TCGA, ICGC, GEO, and CPTAC datasets was combined with single-cell transcriptomic profiling, survival and Cox regression modeling, immune infiltration estimation, and in vitro loss- and gain-of-function experiments in ccRCC cell lines. **Results:** PON2 was markedly elevated in ccRCC tissues, predominantly localizing to malignant epithelial and endothelial cells as determined by single-cell transcriptomic profiling. High PON2 expression correlated with advanced T/M stages and independently predicted poor prognosis. Functional experiments showed that PON2 silencing was associated with reduced tumor cell proliferation, invasion, and glycolysis, while rescue experiments using wild-type—but not S311C mutant—PON2 partially restored these phenotypes. PON2 expression was associated with glycolysis-related metabolic changes and CEBPB-related expression of downstream glycolytic targets, including LDHA and PGK1. Furthermore, PON2-high tumors showed transcriptomic features consistent with an immunosuppressive microenvironment, with higher computational estimates of CD8^+^ T-cell infiltration accompanied by elevated immune checkpoint expression (PD-L1, CTLA4) and higher estimated abundance of immunosuppressive populations (Tregs, MDSCs). **Conclusions:** Collectively, our findings identify PON2 as a candidate biomarker associated with ccRCC progression and CEBPB-related glycolysis and features consistent with an immunosuppressive microenvironment.

## Simple Summary

Clear cell renal cell carcinoma is the most common form of kidney cancer, and patients with advanced disease often stop responding to targeted drugs and immunotherapy. Better markers are needed to predict outcome and explain this resistance. We studied paraoxonase 2, a protective enzyme found inside cells, combining large public patient datasets with laboratory experiments in kidney cancer cells. Tumors contained much more of this enzyme than normal kidney tissue, and patients with high levels survived for a shorter time. Lowering the enzyme made cancer cells grow, move and consume sugar more slowly. Restoring the normal enzyme partly reversed these changes, whereas a version carrying a single amino-acid change did not. Tumors rich in the enzyme also contained more immune cells but, at the same time, more molecules that switch those immune cells off. Paraoxonase 2 may therefore be a useful marker of tumors in which altered metabolism and immune-suppressive features occur together.

## 1. Introduction

Trailing only prostate and bladder malignancies, renal cell carcinoma (RCC) represents a substantial burden within male urological cancers [1]. Recent epidemiological data show that kidney cancer makes up roughly 2% of global cancer incidence and fatality. In men, the incidence is about twice that of women [2,3]. As the dominant histological variant, clear cell renal cell carcinoma (ccRCC) comprises an estimated 70–80% of all RCC diagnoses [4,5]. The current clinical treatments for renal clear cell carcinoma include surgical resection, targeted therapy, and immunotherapy. Advanced ccRCC is less responsive to traditional radiotherapy and chemotherapy. The advent of targeted agents (e.g., VEGFR inhibitors) and immune checkpoint blockade has undeniably transformed the prognostic landscape for advanced patients. Nonetheless, the durable efficacy of these modalities is frequently compromised by the emergence of therapeutic resistance [6,7], driven largely by profound tumor heterogeneity and dynamic metabolic shifts within the tumor microenvironment (TME) [8,9,10]. Therefore, identifying novel biomarkers that link metabolic dysregulation to therapeutic response is of great clinical significance.

Tumor cellular metabolism is fundamentally characterized by an anomalous reliance on aerobic glycolysis, a metabolic feature commonly referred to as the Warburg effect. This fundamental metabolic rewiring extends far beyond mere energy production, actively governing critical facets of tumorigenesis including cell proliferation, invasive capacity, metastatic dissemination, and the modulation of immune responses [11,12]. ccRCC is known as a “metabolic disease model” because of its unique metabolic characteristics. VHL gene inactivation activates the HIF-α pathway, which drives angiogenesis and enhances glycolysis [13]. More importantly, this enhanced glycolytic output invariably leads to massive extracellular lactate accumulation and profound acidification of the TME. This hostile metabolic landscape simultaneously fuels the bioenergetic requirements of rapidly dividing tumor cells and fosters an immunosuppressive milieu. This is achieved by crippling the effector functions of cytotoxic T lymphocytes [14] while reciprocally favoring the expansion of immunosuppressive regulatory T cells [15]. Despite these observations, the precise molecular conduits orchestrating the interplay between elevated glycolytic activity and immunological subversion in ccRCC have yet to be delineated.

Paraoxonase 2 (PON2) is a widely expressed intracellular antioxidant protein involved in cellular redox defense, mitochondrial homeostasis, and apoptosis regulation [16,17]. The function of PON2 is closely linked to tumor initiation and progression. Studies indicate that PON2 is overexpressed in different malignant tumors and is significantly associated with tumor progression and resistance to treatment. For instance, heightened PON2 expression in gastric cancer has been associated with poorer overall survival [18], while altered PON2 expression and function have also been reported in bladder cancer tissues and cell models [19]. Similarly, in triple-negative breast cancer, aberrant PON2 signaling has been associated with malignant progression and may support cellular defense mechanisms against chemotherapeutics [20]. Although PON2 has been implicated in modulating glucose transporter expression in pancreatic cancer [21], whether it regulates the unique glycolytic phenotype of ccRCC and whether this regulation relies on its enzymatic activity remain unknown. Furthermore, its potential role in shaping the immune landscape of ccRCC has not been explored.

To address these critical knowledge gaps, the present study embarked on a systematic exploration of PON2’s expression profiles, prognostic implications, and functional repertoire in ccRCC. By deploying an integrative approach combining high-throughput transcriptomic analyses with in vitro molecular assays, we sought to characterize the association between PON2, CEBPB, and tumor glycolysis. Ultimately, this research aims to characterize the association of PON2 with an immunosuppressive TME and its potential relevance to ccRCC progression.

## 2. Materials and Methods

### 2.1. Study Period

This study was conducted between January 2025 and February 2026.

### 2.2. Data Sources and Preprocessing

Raw transcriptomic data and associated clinical annotations for a pan-cancer cohort were systematically retrieved from Xena (https://xena.ucsc.edu/, accessed on 5 January 2025), specifically focusing on The Cancer Genome Atlas (TCGA), from which 539 TCGA-KIRC tumors and 72 adjacent normal tissues were analysed. To ensure comparability across samples, we transformed the raw fragments per kilobase of exon model per million mapped fragments (FPKM) into transcripts per kilobase of exon model per million mapped reads (TPM) values, and log_2_(TPM + 1) normalization was applied. Additionally, protein-level expression data for renal tumors and adjacent normal tissues were acquired from the CPTAC (https://cptac-data-portal.georgetown.edu/, accessed on 15 January 2022) repository (*n* = 194).

To further elucidate the mRNA and protein level landscape of PON2, additional datasets from the ICGC (RECA-EU; 91 tumors and 45 adjacent normal tissues with RNA sequencing), multiple GEO series (GSE40435, *n* = 202; GSE53757, *n* = 120; GSE36895, *n* = 52; and GSE15641, *n* = 55), and the CZ_Protein cohort (*n* = 34) were integrated. The independent E-MTAB-1980 ccRCC expression and survival cohort (*n* = 101) was obtained from ArrayExpress/EMBL-EBI (https://www.ebi.ac.uk/biostudies/arrayexpress, accessed on 5 January 2025) and used for external validation of the association between PON2 expression and overall survival. PON2 expression values and clinical annotations were extracted according to the dataset metadata, and patients with available survival information were dichotomized into high- and low-PON2 groups using the median expression value for Kaplan–Meier analysis. Only ccRCC tumor samples and non-tumor kidney samples were analysed; other renal tumor types and xenograft samples present in some of these series were not included. Patients whose clinical annotation was missing were omitted from the analyses that involve clinical variables.

For single-cell resolution analysis, transcriptomic data for human malignancies (GSE159115, *n* = 8) were retrieved from the TISCH website. This database provides preprocessed expression matrices along with dimensionality reduction, clustering, and cell-type annotations. We downloaded both the gene expression matrix and metadata and performed visualization in R using the Seurat package (v4.0.5). The two-dimensional spatial distribution of cells was presented via Uniform Manifold Approximation and Projection (UMAP). Following the categorical metadata provided by the TISCH database, the heterogeneous cell landscape was partitioned into eight distinct populations. These included both malignant and epithelial compartments, endothelial and pericyte subsets, as well as several immune-related lineages such as erythroblasts, plasma cells, CD8^+^ T lymphocytes, and the monocyte/macrophage fraction. To visualize the expression density of PON2, the ‘Nebulosa’ R package (v1.18.0) was used to generate feature density maps. Moreover, dot plots were created using the DotPlot function to evaluate both the frequency and mean intensity of PON2 expression across distinct cell subsets. Additionally, PON2 expression was specifically compared between epithelial and malignant cells using violin plots. Pairwise comparisons were evaluated through the Wilcoxon rank-sum test, defining *** p<0.001 as the threshold for statistical significance.

### 2.3. The CZ_Protein Cohort

The CZ_Protein cohort consists of 17 paired specimens of ccRCC tumor tissue and matched adjacent non-tumor kidney tissue, collected at Changzhou Second People’s Hospital in January 2025. Patients were eligible if ccRCC had been confirmed by histopathological diagnosis. Tissue was snap-frozen after resection and stored at −80 °C until analysis. Protein abundance was determined by data-independent acquisition (DIA) quantitative proteomics, performed by OE Biotech (Shanghai, China). These are the specimens covered by the ethical approval cited in the Institutional Review Board Statement.

### 2.4. Functional Enrichment and Regulatory Network Construction

To dissect the biological signaling dynamics, Gene Set Enrichment Analysis (GSEA) was performed using GSEA software (v4.3.3, Broad Institute, Cambridge, MA, USA) utilizing reference sets from MSigDB, specifically the c2.cp.kegg.v7.5.1 and h.all.v7.2 Hallmark collections. Based on the median PON2 abundance, samples were bifurcated into high- and low-expression cohorts. We scrutinized the enrichment status across multiple functional modules, encompassing N-Glycan biosynthesis (KEGG) alongside several Hallmark pathways: Glycolysis, IL6-JAK-STAT3 signaling, and the inflammatory/interferon-gamma responses. One thousand permutations were used. Immune cell infiltration was estimated with the TIMER and MCP-counter algorithms and by single-sample GSEA (ssGSEA) of 28 immune cell signatures, as implemented in the IOBR R package (v2.0.0).

### 2.5. Therapy-Related Gene Signature Analysis

Therapy-related gene signatures were taken from the published collection assembled by Hu et al. [22], which brings together gene sets associated with the response to immune checkpoint blockade, chemotherapy, anti-angiogenic therapy and targeted therapies. Enrichment scores for each signature were computed for every TCGA-KIRC sample from the log_2_(TPM + 1) expression matrix using the GSVA R package (v1.52.3).

### 2.6. Cell Culture and Model Construction

Normal human renal cortical proximal tubular epithelial cells (HK2, GNHu47) and ccRCC cell lines (786-O, TCHu186; Caki-1, TCHu135; OSRC-2, TCHu40; ACHN, TCHu199; Caki-2, TCHu251; 769-P, TCHu215) were obtained from the Shanghai Cell Bank of the Chinese Academy of Sciences. The populations were maintained under a humidified atmosphere of 5% CO_2_ at 37 °C, utilizing specialized growth media (McCoy’s 5A or RPMI 1640) enriched with 10% fetal bovine serum. PON2 was silenced using a short hairpin RNA directed against nucleotides 600–620 of the PON2 transcript (RefSeq NM_000305.3; 5′-GCACATTTCTATGCCACAAAT-3′). This site lies within the coding sequence, spanning codons 175–181, and not in the 3′UTR. Lentivirus (LV-shPON2) was packaged and used to infect cells at an MOI of 20, followed by selection of stable clones with puromycin (5 µg/mL).

For the reconstitution experiments, wild-type PON2 (PON2^#WT^) and the S311C mutant (PON2^#MT^) were expressed from pTSBX-CMV-PON2(NM_000305)-3xFLAG-EF1-BSD-T2A-mCherry, which carries the PON2 open reading frame with a C-terminal 3 × FLAG tag under a CMV promoter. Because the shRNA target site lies within the coding sequence, both reconstitution constructs were rendered shRNA-resistant by introducing three synonymous substitutions at the wobble positions of His176, Tyr178 and Thr180 (CAT to CAC, TAT to TAC and ACA to ACT). These substitutions create three mismatches at positions 6, 12 and 18 of the 21-nucleotide target site, while leaving the encoded protein sequence unchanged, so that the exogenous transcript escapes shRNA-mediated silencing. The matched empty-vector construct (pTSBX-CMV-MSC-EF1-BSD-T2A-mCherry) served as the control. All three plasmids were verified across the insert by bidirectional Sanger sequencing. Stable clones were selected using Blasticidin S (5 µg/mL).

### 2.7. Proliferation Ability Detection

To evaluate the proliferative potential of the cells, CCK-8 and EdU assays were performed. For the CCK-8 experiment, cells maintained in the logarithmic growth phase were harvested and distributed into 96-well culture plates at a density of 1000 cells per well. To minimize peripheral evaporation (commonly termed the “edge effect”), the outermost wells were filled with 100 µL of PBS. Following incubation under standard conditions (37 °C, 5% CO_2_) for predefined intervals (12, 24, 36, 48, and 72 h), each well was supplemented with 10 µL of Cell Counting Kit-8 reagent. Absorbance values at a wavelength of 450 nm were subsequently recorded by a Synergy LX microplate reader (BioTek Instruments, Winooski, VT, USA) after an additional 1 h incubation.

For the EdU incorporation assay, $3\times 10^{5}$ cells (logarithmic phase) were seeded into each well of a 6-well plate and allowed to adhere for 24 h. Proliferating cells were subsequently identified using the BeyoClick™ EdU-555 Detection Kit (Beyotime Biotech Inc., Shanghai, China). Briefly, a 20 µM (2×) EdU working solution was prepared in a 15 mL centrifuge tube and pre-warmed to 37 °C. Each well received 1 mL of this solution, followed by a 2 h incubation. Post-incubation, cells were fixed with paraformaldehyde for 15 min, then rinsed three times using QuickBlock™ blocking solution (P0260; Beyotime). Immunostaining permeabilization solution (P0097, Beyotime Biotech Inc., China) was added, discarded after 15 min, and washed twice. The Click reaction solution was prepared according to the instructions, 500 µL was added to each well, and the plate was incubated in the dark for 30 min. Hoechst 33342 was diluted at 1:1000, 1 mL was added to each well, incubated in the dark for 10 min, and washed three times. Cell fluorescence was observed under an IX71 inverted fluorescence microscope equipped with a DP73 camera (Olympus Corporation, Tokyo, Japan), and photographs were taken for record.

### 2.8. Cell Migration Analysis

Cells in the logarithmic growth phase were selected, and a cell suspension containing $1\times 10^{4}$ cells in every 200 µL of suspension was prepared. Then, 200 µL of the freshly prepared cell suspension was added to each Transwell cell culture chamber (8 µm, Corning Inc., Corning, NY, USA); the culture medium was discarded after 36 h of incubation, followed by washing with PBS. Next, 400 µL of 4% paraformaldehyde solution was added to a new 24-well plate, and the washed chambers were placed into the wells for fixation for 20 min. The chambers were removed and washed twice with PBS. Then, 400 µL of 0.1% crystal violet solution (Beyotime Biotech Inc., China) was added to the new 24-well plate, and the chambers were placed into the wells for staining for 8 min. The chambers were removed and washed with PBS, and after drying, the inside of the chambers was wiped with a cotton swab to clean the cells. Cell staining was observed under the IX71 microscope (Olympus) and photographs were taken for record. ImageJ software (v1.54p, National Institutes of Health, Bethesda, MD, USA) was used for counting stained cells, and statistical analysis was performed on the experimental data.

### 2.9. Cell Invasion Analysis

To assess invasive capacity, Transwell chambers received a 50 µL Matrigel coating, which had been diluted at a 1:8 ratio in RPMI 1640 medium. A cell suspension containing $2\times 10^{4}$ cells in a volume of 200 µL suspension was prepared, and the remaining experimental steps were the same as those in the migration analysis.

### 2.10. Glycolysis Rate Measurement

Cells were seeded at 150,000 per well in six-well plates in RPMI 1640 containing 10 percent fetal bovine serum and cultured for 48 h. The medium was then collected and centrifuged to remove cell debris, and the supernatant was taken for measurement. Cell-free medium incubated in parallel served as the baseline, so that glucose consumption is reported as the difference between that baseline and the sample. According to the instructions in the glucose (GLU) kit (A154-2-1, Nanjing Jiancheng Bioengineering Institute, Nanjing, China), each reagent was added to a 96-well plate and incubated at 37 °C for 10 min. Absorbance (A) was subsequently recorded at 505 nm using a microplate reader. Glucose consumption was calculated using the formula: glucose concentration (mmol/L) = (A_measured_ − A_blank_)/(A_standard_ − A_blank_) × C_standard_ × sample dilution factor N, with a standard solution concentration of 5.55 mmol/L.

Using the above method, the supernatant was taken and, according to the instructions in the pyruvate kit (A081-1-1, Nanjing Jiancheng Bioengineering Institute, China), each reagent was added to a 96-well microplate; the mixture was allowed to stand at ambient temperature for 5 min, and the absorbance value A of each tube was measured at 505 nm. The amount of pyruvate produced was calculated using the formula: pyruvate concentration in the liquid sample (µmol/mL) = (A_measured_ − A_blank_)/(A_standard_ − A_blank_) × C_standard_, with a standard solution concentration of 0.2 mmol/L.

Using the above method, the supernatant was taken and, according to the instructions in the L-lactic acid (LAC) measurement kit (A019-1-1, Nanjing Jiancheng Bioengineering Institute, China), each reagent was added to a 96-well microplate. The OD value was measured at 546 nm before adding reagent two, and after incubating at 37 °C for 5 min with reagent two, the absorbance value A2 was measured again at 546 nm. The amount of L-lactic acid produced was calculated using the formula: L-lactic acid concentration (mmol/L) = (ΔA_measured_ − ΔA_blank_)/(ΔA_standard_ − ΔA_blank_) × C_standard_ × sample dilution factor N, where ΔA = A2 − A1, with a standard solution concentration of 3 mmol/L.

All three measurements were normalized to the total protein content of the corresponding well, determined with a BCA assay on the cell lysate collected at the same time, and are reported per unit protein. Because PON2 knockdown also reduces proliferation, this normalization removes the contribution of differences in cell number between the groups. These are extracellular metabolite measurements rather than a direct measurement of glycolytic flux.

### 2.11. Quantitative Real-Time PCR

Total RNA isolation was performed following the manufacturer’s protocol provided with the Total RNA Isolation Kit. To determine relative transcript abundance, the isolated RNA was subsequently synthesized into complementary DNA (cDNA) using HiScript II Q RT SuperMix (Vazyme Biotech Co., Ltd., Nanjing, China). Quantitative PCR detection was then performed utilizing the QuantiNova SYBR Green PCR Kit (QIAGEN, Hilden, Germany), with the amplification process executed on a QuantStudio 5 Real-Time PCR System (Applied Biosystems, Thermo Fisher Scientific, Waltham, MA, USA). Primer sequences employed throughout this study are provided in Table S1.

### 2.12. Western Blot

Total protein fractions were isolated utilizing RIPA lysis buffer (Beyotime Biotech Inc., China), and their concentrations were determined employing a BCA protein assay kit (Beyotime). Following resolution via SDS-PAGE electrophoresis, the separated proteins were electrotransferred onto PVDF membranes (Merck Millipore, Burlington, MA, USA). Membranes were probed with target-specific primary antibodies overnight at 4 °C, followed by incubation with species-matched HRP-linked secondary antibodies at a 1:5000 dilution, as specified below. All primary antibodies were obtained from Proteintech (Wuhan, China) and were used at the following catalogue numbers and working dilutions: PON2 (14379-1-AP, rabbit polyclonal, 1:500), CEBPB (66649-1-Ig, mouse monoclonal, 1:3000), JUN (66313-1-Ig, mouse monoclonal, 1:20,000), JUNB (10486-1-AP, rabbit polyclonal, 1:500), PGAM1 (67470-1-Ig, mouse monoclonal, 1:20,000), PFKP (68129-1-Ig, mouse monoclonal, 1:20,000), HK1 (68419-1-Ig, mouse monoclonal, 1:20,000), PGK1 (68035-1-Ig, mouse monoclonal, 1:20,000), LDHA (66287-1-Ig, mouse monoclonal, 1:1000) and β-actin (66009-1-Ig, mouse monoclonal, 1:50,000). The secondary antibodies were HRP-conjugated goat anti-mouse IgG (H+L) (SA00001-1, Proteintech, 1:5000) and HRP-conjugated goat anti-rabbit IgG (H+L) (SA00001-2, Proteintech, 1:5000). For densitometric quantification, the target band was identified according to the expected molecular weight or molecular-weight range specified in the manufacturer’s product information for each antibody, namely PON2 at approximately 39 kDa, CEBPB at 40–45 kDa, JUN at 39 kDa, JUNB at 42 kDa, PGAM1 at 29 kDa, PFKP at 80–86 kDa, HK1 at 100 kDa, PGK1 at 40–45 kDa, LDHA at 32–37 kDa and *β*-actin at 42 kDa, together with its migration relative to the molecular-weight marker run on the same membrane. When additional immunoreactive bands were present, only the band lying within the prespecified expected molecular-weight range was included in the densitometric analysis, and bands outside that range were excluded. The same band-selection criterion was applied consistently across all lanes analyzed for a given target. Protein signals were visualized and detected using the Immobilon™ Western chemiluminescent HRP substrate (Merck Millipore) and an SCG-W2000 PLUS chemiluminescence imaging system (Servicebio, Wuhan, China). Band intensities were quantified by densitometry in ImageJ (v1.54p, National Institutes of Health, Bethesda, MD, USA), with the signal for each target normalized to the β-actin signal of the same lane, and quantification was performed across three independent experiments. Uncropped images of all Western blots are provided in Figures S1–S5.

### 2.13. Statistical Analysis

Bioinformatics workflows were executed within the R version 4.4.1 environment. Continuous variables conforming to a normal distribution were summarized as mean ± standard deviation; otherwise, medians with interquartile ranges were reported. To evaluate clinical outcomes, the “survival” package (v3.8-3) was employed to construct survival trajectories via the Kaplan–Meier method, with inter-group disparities interrogated through the log-rank test. For in vitro experimental data, statistical processing was executed using GraphPad Prism 10.1.2 software. Discrepancies between two cohorts were determined using Student’s *t*-test. All in vitro experiments were performed as three independent biological replicates (n=3), and all bar and line graphs display the mean ± standard deviation (SD). Comparisons among more than two groups were made by one-way analysis of variance followed by Dunnett’s multiple-comparison test against the relevant control group. The CCK-8 time courses, which include both a treatment and a time factor, were analysed by two-way analysis of variance followed by Dunnett’s multiple-comparison test. Cox proportional hazards models, both univariate and multivariate, were employed to assess the independent prognostic relevance of PON2. In both models, histological grade was coded as a binary variable, G1–2 versus G3–4, and clinical stage likewise as stage I–II versus stage III–IV, while age was treated as a continuous variable. Associations between PON2 and the immune-related gene categories summarized in the circular heatmap were assessed by Pearson correlation, whereas associations between PON2 and individual immune checkpoint genes were assessed by Spearman rank correlation. Statistical significance was defined by a *p*-value <0.05.

## 3. Results

### 3.1. Characterization of PON2 Expression Heterogeneity and Cellular Localization in ccRCC

Initial exploration of the TCGA pan-cancer landscape revealed a marked heterogeneity in PON2 expression across diverse human malignancies. Specifically, in the context of ccRCC, PON2 abundance was significantly enriched within tumorous tissues relative to their adjacent normal counterparts (Figure 1A). This finding was corroborated by paired-sample comparisons, which demonstrated a consistent and significant elevation of PON2 levels in ccRCC lesions, and a similar tumor-associated increase was seen in several of the other TCGA cancer types shown in (Figure 1B). To bolster the reliability of these observations, we executed an analysis across multiple validation platforms, including the ICGC, GEO, CPTAC, and CZ_Protein cohorts. Collectively, these multi-omics data confirmed upregulation of both PON2 transcripts and protein levels within ccRCC specimens (Figure 1C–F).

At the single-cell resolution, transcriptomic profiling indicated that the endothelial and malignant epithelial cell compartments were the primary cellular reservoirs for PON2 expression. Furthermore, a refined comparative analysis between normal and transformed epithelial populations revealed that PON2 was preferentially and significantly overexpressed in the malignant subset (Figure 1G–J).

Clinicopathological correlation analysis indicated that PON2 expression was significantly and positively associated with clinical stage, especially T/M stage in those with ccRCC, whereas no significant associations were observed with sex, histological grade, or N stage (Figure 1K), suggesting a potential link between PON2 expression and tumor proliferation and metastatic phenotypes.

These findings were further reinforced by in vitro evaluations, showing that both mRNA and protein abundance of PON2 were heightened across a panel of six RCC cell lines (786-O, OSRC-2, Caki-2, ACHN, Caki-1, and 769-P) compared with the HK2 cell line (Figure 1L). PON2 protein was quantified by densitometry and normalized to β-actin in the same lane across three independent experiments (Figure 1L).

### 3.2. Prognostic Significance of PON2 as an Independent Predictor of Clinical Outcomes in ccRCC

Survival trajectory assessments within the TCGA cohort showed that elevated PON2 expression was associated with a poorer clinical outlook. Specifically, patients in the high-PON2 group experienced markedly abbreviated overall survival (OS) relative to their low-expression counterparts (Figure 2A). A parallel and significant diminution in progression-free survival (PFS) was also observed in cases with high PON2 abundance (Figure 2B). These findings were confirmed in the E-MTAB-1980 cohort (Figure 2C). Moreover, Cox regression analyses at both univariate and multivariate levels identified high PON2 expression as an independent unfavorable prognostic indicator for ccRCC patients (Figure 2D–G).

### 3.3. The Interplay Between PON2 Expression and the Immunogenic Landscape Within the ccRCC Microenvironment

To dissect the immunological implications of PON2 in ccRCC, we divided the TCGA-KIRC cohort into two subgroups according to median PON2 expression and subsequently performed GSEA. Our findings demonstrated that the PON2-high group was characterized by a significant enrichment of several pathways governing inflammatory dynamics and immune mobilization. Specifically, Hallmark signatures including the IL6-JAK-STAT3 signaling cascade, interferon-gamma response, and inflammatory response were significantly enriched (Figure 3A–C). These data indicate an association between elevated PON2 levels and IFN-γ-associated immune signatures and IL6-JAK-STAT3 signaling in the ccRCC context.

Furthermore, we interrogated the associations between PON2 and various immune regulatory dimensions. The circular heatmap revealed broad associations between PON2 and multiple immune functional modules in the TCGA-KIRC cohort (Figure 3D). Specifically, PON2 exhibited an overall positive correlation with the chemokine/chemokine receptor axes (CXCL/CCL and CXCR/CCR), suggesting that elevated PON2 may be accompanied by immune cell recruitment-related signals. Meanwhile, PON2 also showed consistent positive associations with antigen presentation-related molecules (e.g., B2M and the HLA family), indicating that increased PON2 expression may coincide with antigen-presentation-related expression. In addition, multiple immune-stimulatory/immune-modulatory molecules (such as TNFSF/TNFRSF-related molecules and certain co-stimulatory factors) were also significantly positively correlated with PON2 expression, collectively supporting that high PON2 expression is coupled with a molecular phenotype of “broadly altered immune regulatory expression” (Figure 3D). Notably, when extending the analysis to other urologic tumor cohorts (e.g., BLCA, KIRP, KICH, PRAD), an overall consistent correlation trend between PON2 and the immune-related modules above was observed across multiple cohorts, although the correlation strength varied by cancer type. In contrast, correlations were generally weaker in the TGCT cohort, with some indicators showing negative or opposite-direction trends (Figure 3D), suggesting that the association between PON2 and immune features may be tumor type-specific.

Given the clinical relevance to immune checkpoint blockade (ICB), we specifically scrutinized the correlation between PON2 and inhibitory molecules. Spearman correlation analysis demonstrated that in ccRCC, PON2 expression was significantly positively correlated with PD-L1 (CD274), PD-1 (PDCD1), and CTLA4. Similarly, in other urologic tumor cohorts (e.g., BLCA, KIRP, KICH, PRAD), PON2 was predominantly positively correlated with these immune checkpoint molecules, although the correlation strength differed across cancer types. By comparison, correlations were weaker in the TGCT cohort, and some checkpoint molecules showed negative or opposite-direction trends (Figure 3E–G). These results indicate that increased PON2 expression is often accompanied by concomitant upregulation of immune checkpoints, suggesting that high PON2 expression is associated with an immunosuppressive TME phenotype.

Finally, a quantitative assessment of 28 distinct immune cell lineages revealed that PON2 expression was associated with the estimated infiltration landscape (Figure 3H). Crucially, while PON2 positively correlated with effector subsets like activated CD4^+^ T and NK cells, it was also associated with higher estimated abundance of immunosuppressive components, including regulatory T cells (Tregs) and myeloid-derived suppressor cells (MDSCs). Collectively, these results suggest that the co-occurrence of an inflamed phenotype and immunosuppressive cell estimates may reflect a highly dysfunctional and suppressive immunological niche in ccRCC.

### 3.4. High PON2 mRNA Level Is Correlated with Enhanced Infiltration of Immune Cells and an Immunosuppressive TME

While our initial ssGSEA results hinted at a link between PON2 and the immune landscape, we further evaluated these findings using the TIMER and MCP-counter computational frameworks. Analysis via the TIMER algorithm revealed a higher estimated abundance of immune cells in high-PON2 tumors, particularly within the CD8^+^ T-cell, dendritic cell, and macrophage populations, suggesting an immune-infiltrated state with antigen-presentation-related features (Figure 4A). Consistently, the MCP-counter results further indicated significantly increased infiltration of cytotoxic lymphocytes and T cells in the PON2-high group, while NK cells, the monocyte lineage, and myeloid dendritic cells also showed a trend toward higher infiltration. Notably, beyond immune cells, endothelial cell and fibroblast scores were also increased in the PON2-high group, implying that PON2 expression differences were accompanied by changes beyond immune-cell estimates, including differences in vascular and stromal scores (Figure 4B).

Regarding the assessment of immune inflammatory status, PON2 expression displayed a significant positive trend toward T-cell inflamed score (TIS) (R=0.2, $p=4.5\times 10^{-6}$), indicating that elevated PON2 is associated with an inflamed tumor immune microenvironment (Figure 4C). Further correlation network and heatmap analyses demonstrated broad positive associations between PON2 and multiple immune effector molecules. In terms of T-cell cytotoxic function and the Th1 immune axis, PRF1, GZMB, GZMA, CCL5, CD8A, IFNG, TBX21, and CXCL10/CXCR3 all showed significant positive correlations with PON2, suggesting that high PON2 expression co-occurs with cytotoxic T-cell-related and IFN-γ-associated signatures. Meanwhile, PON2 also showed significant correlations with various myeloid cell-related markers, including CSF1R, CYBB, MS4A6A, MARCO, and C1QA, indicating that myeloid infiltration-related features are also associated with the immune microenvironment in the context of elevated PON2 (Figure 4D).

To visually depict the expression patterns of PON2-associated immune effector genes, we categorized immune effector molecules significantly correlated with PON2 by cell type (e.g., CD8 T cells, Th1 cells, NK cells, dendritic cells, and macrophages). The heatmap revealed that, in PON2-high samples, genes related to cytotoxic effector function and the Th1 axis (e.g., PRF1, GZMB, CXCL10, IFNG, TBX21) as well as markers associated with myeloid cells/antigen presentation (e.g., CSF1R, C1QA, MARCO) were broadly upregulated, further supporting a positive association between PON2 and an inflamed microenvironment (Figure 4G). A systematic evaluation of PON2 against 21 immune checkpoint molecules revealed that PON2 was significantly correlated with the expression of numerous checkpoint genes, which also displayed an overall upregulation trend in the PON2-high group (Figure 4E,F). This indicates that, although T-cell inflammatory signals and effector molecules are globally enhanced in the PON2-high context, the concomitant upregulation of immune checkpoints is consistent with an immunosuppressive tumor microenvironment. Furthermore, based on previously reported therapy-related gene sets [22], we conducted an exploratory assessment of therapy-related expression signatures and found that the PON2-high group showed higher expression trends for signatures related to chemotherapy, EGFR-targeted therapy, and anti-angiogenic therapy (Figure 4H), which warrants further investigation in independent treatment-response cohorts.

### 3.5. Knockdown of PON2 Attenuates the Malignant Behavior of ccRCC Cells In Vitro

To determine whether PON2 contributes to ccRCC malignant progression, stable PON2-depleted 786-O and OSRC-2 cell models were constructed via lentiviral transfection. RT-qPCR and Western blot analyses revealed that PON2 expression was efficiently suppressed in both cell lines (Figure 5A,B). CCK-8 cell viability assays and EdU incorporation assays consistently demonstrated that, compared with the negative control (NC) group, PON2 knockdown inhibited the proliferative capacity of both cell lines, manifested as decreased viability readings and a substantially lower fraction of EdU-positive cells (Figure 5C–F). Transwell assays further revealed that PON2 knockdown significantly diminished the migratory and invasive capacities of 786-O and OSRC-2 cells, with considerably fewer cells traversing the membrane in the PON2^#sh^ group compared with NC controls (Figure 5G,H).

### 3.6. Reconstitution with Wild-Type but Not S311C PON2 Restores Malignant Phenotypes in ccRCC Cells

PON2 (paraoxonase 2) is a member of the paraoxonase family and functions as an important esterase, playing key roles in various biological processes, including anti-inflammatory and antioxidant responses. To examine whether the S311C substitution alters the association of PON2 with malignant phenotypes of ccRCC, we overexpressed wild-type PON2 (PON2^#WT^) and an S311C mutant (PON2^#MT^) in PON2-knockdown cells (PON2^#sh^) (Figure 6A). Following Blasticidin S selection, stable cell lines were established and validated by RT-qPCR and Western blot analyses (Figure 6B,C), resulting in three groups: PON2^#Vec^ (PON2^#sh^ cells transduced with the empty vector), PON2^#WT^, and PON2^#MT^. Proliferation analysis via CCK-8 showed higher viability in PON2^#WT^-reconstituted cells compared with PON2^#Vec^ and PON2^#MT^ counterparts in both 786-O and OSRC-2 lines, with PON2^#Vec^ and PON2^#MT^ groups showing comparable viability (Figure 6D,E). In addition, migration and invasion analyses using Transwell chambers indicated that the PON2^#WT^ group exhibited the most prominent recovery of migratory and invasive phenotypes (Figure 6F,G). Collectively, reconstitution with wild-type, but not S311C, PON2 restored these in vitro phenotypes.

### 3.7. PON2-Associated Glycolysis and CEBPB Expression

Pathway-level analysis via GSEA demonstrated that glycolysis-associated gene sets were markedly enriched in the PON2-high cohort (Figure 7A,B). To further evaluate the association between PON2 and glycolysis-related metabolism, we measured glucose consumption as well as pyruvate and lactate production in PON2-knockdown 786-O and OSRC-2 cell lines. Relative to NC controls, all glycolysis-related metabolic parameters were significantly reduced (Figure 7C–E).

Bioinformatics analysis further demonstrated that, among renal cancer samples with different levels of PON2 expression, the expression levels of multiple key glycolytic enzymes, including PGAM1, PFKP, HK1, PGK1, and LDHA, were significantly different (Figure 7G). Subsequent analysis of upstream transcription factors associated with these enzymes revealed that, among CEBPB, JUNB, and JUN, only CEBPB exhibited significant differential expression across renal cancer samples with varying PON2 expression levels (Figure 7F), identifying CEBPB as a candidate transcription factor whose expression covaries with that of PON2.

To examine this association further, RT-qPCR and Western blot analyses were performed in the previously established 786-O and OSRC-2 cell models. PON2 depletion resulted in significant downregulation of CEBPB along with multiple glycolytic enzymes (PGAM1, PFKP, HK1, LDHA, and PGK1) at both mRNA and protein levels (Figure 7H–J). Functional rescue experiments further showed that PON2^#WT^ partially restored the glycolysis-related metabolic measurements, whereas PON2^#MT^ did not produce a comparable change (Figure 7K–M). These observations were corroborated at the molecular level, as RT-qPCR and immunoblotting verified that only PON2^#WT^ partially recovered the reduced expression of CEBPB and the glycolytic enzymes examined (Figure 7N–P).

It is worth noting that JUN and JUNB failed to demonstrate any significant relationship with PON2 expression, collectively suggesting that PON2 expression is associated with CEBPB and glycolysis-related genes in a manner influenced by the S311C substitution.

## 4. Discussion

This study, by integrating bioinformatics analyses with experimental validation, characterizes the association between paraoxonase 2 (PON2) and the progression of ccRCC and highlights its clinical significance. PON2 is markedly upregulated in ccRCC, and its expression is positively linked to the clinical stage as well as T and M classifications. In multivariate Cox modeling, high PON2 expression emerged as an independent prognostic factor for adverse outcomes in ccRCC. In vitro functional validation revealed that PON2 silencing was associated with reduced proliferative, migratory, and invasive behaviors in ccRCC cells, and that reconstitution with wild-type, but not S311C, PON2 restored these phenotypes. PON2 expression was associated with glycolysis-related metabolic changes and CEBPB expression. Our data also disclosed that elevated PON2 is associated with transcriptomic features of an immunosuppressive tumor milieu, with concurrent immune checkpoint upregulation. Collectively, these findings suggest that PON2 represents not only a novel prognostic biomarker for ccRCC but also a key molecular feature associated with metabolic reprogramming and an immunosuppressive microenvironment.

In recent years, PON2 has garnered attention as an endogenous antioxidant enzyme due to its aberrant expression in various malignancies. Previous studies have shown significant upregulation of PON2 in cancer cells such as gastric cancer [18], bladder cancer [19], triple-negative breast cancer [20], and advanced oral squamous cell carcinoma [23]. Consistent with these studies, our research found PON2 to be significantly upregulated in ccRCC. Additionally, our study utilized single-cell sequencing to clarify that PON2 is mainly localized in cancer cells and endothelial cells in clear cell renal carcinoma, suggesting that PON2 may be associated with tumor metabolism and the vascular microenvironment; this possibility warrants further study [24,25,26].

Clinicopathological analysis uncovered a significant positive association between PON2 abundance and both T and M classifications. Thus, we examined the association of PON2 with cell proliferation and metastatic phenotypes. In lung cancer research, the loss of PON2 expression significantly inhibited the proliferation of lung cancer cells and caused cell cycle arrest at the G1 phase, indicating that PON2 may promote tumor cell proliferation by regulating important genes and signaling pathways of the cell cycle [27]. In triple-negative breast cancer (TNBC), reduced PON2 levels significantly decreased cancer-cell proliferation and increased chemosensitivity [20]. PON2-dependent promotion of cell migration has also been observed in bladder cancer cells in vitro [28], supporting a broader association between PON2 and tumor-cell motility. In oral squamous cell carcinoma, the expression level of PON2 is also closely related to tumor invasiveness and prognosis [23]. Our study shows that PON2 expression is associated with proliferative and metastatic phenotypes in clear cell renal cell carcinoma (ccRCC). Specifically, PON2 knockdown was associated with reduced proliferative, migratory, and invasive potential of RCC cells in vitro.

In an in vitro study of acetylcholinesterase-inhibiting drugs used in Alzheimer’s disease, PON2 esterase activity was associated with the hydrolysis of donepezil hydrochloride and pyridostigmine bromide, and the S311C polymorphic variant altered the measured esterase activity [29]. In a diabetic retinopathy model, a glycation-resistant K70A PON2 variant showed enhanced antiglycation effects and reduced intracellular oxidative stress, endoplasmic reticulum stress, and inflammatory responses [30]. Evidence regarding the functional importance of residue 311 is not uniform. One study found that the native Ser/Cys311 polymorphism was not critical for PON2 hydrolytic, antioxidant or anti-apoptotic activity [31], whereas another reported that replacing Ser311 with cysteine preserved protein expression and subcellular localization but altered glycosylation and reduced lactonase activity [32]. Our study constructed the PON2 S311C mutant and found that wild-type, but not S311C, PON2 restored the phenotypes examined. We did not, however, measure lactonase or esterase activity in our cells. The difference between the two constructs is therefore consistent with a contribution of PON2 enzymatic activity, but it does not exclude effects of the substitution on protein stability, glycosylation, subcellular localization or protein interactions. Discriminating between these possibilities will require direct measurement of PON2 enzymatic activity in the reconstituted cells.

Glycolysis is a crucial metabolic pathway in tumor development. Glycolysis supplies vital energy and building blocks for tumor cells, and it is also linked to tumor growth, spread, and immune evasion [33]. For example, in hepatocellular carcinoma, studies have shown that high glycolytic activity is significantly associated with poor prognosis, and by affecting the metabolic state of cells, it regulates the tumor microenvironment, thereby promoting tumor progression [34,35]. Previous studies indicate that PON2 increases glycolysis in pancreatic cancer cells by boosting GLUT1 expression [21]. PON2 knockdown significantly reduced LDHA expression. More broadly, dysregulated glycolysis has been linked to malignant progression and remodeling of the tumor microenvironment [36,37]. CEBPB is a transcription factor widely reported in cancer, belonging to the bZIP transcription factor family [38]. In colon cancer, CEBPB promotes tumor progression by enhancing aerobic glycolysis [39]. Our study further indicates that PON2 expression is associated with ccRCC progression and CEBPB-related glycolysis. Interestingly, other bZIP family members like JUN and JUNB did not correlate with PON2, indicating that the association between PON2 and bZIP family transcription factors is selective rather than general.

Another major finding of this study is the complex interplay between PON2 and the tumor immune microenvironment. Our immune infiltration analyses revealed that high PON2 expression is positively correlated with CD8^+^ T-cell infiltration, consistent with an inflamed immune phenotype [40]. This correlation needs to be read in the context of ccRCC, which is unusual among solid tumors in being heavily T-cell infiltrated. Among 592 advanced ccRCC tumors treated with PD-1 blockade, only 27 percent showed a non-infiltrated phenotype, and the degree of CD8^+^ T-cell infiltration was not associated with clinical response [41]. Mass cytometry of 73 ccRCC patients showed that the composition of the macrophage and T-cell infiltrate, rather than its magnitude, correlated with progression-free survival [42], and in a series of 756 ccRCC cases, high infiltration by cytotoxic T cells, macrophages and granulocytes was associated with shorter cancer-specific survival [43]. Single-cell profiling has further shown that terminally exhausted CD8^+^ T cells and M2-like macrophages accumulate together as the disease advances [44]. However, PON2 expression was simultaneously strongly associated with elevated levels of immune checkpoint molecules (PD-L1, CTLA4, and PD-1) and enrichment of immunosuppressive cell populations, including regulatory T cells (Tregs) and myeloid-derived suppressor cells (MDSCs). Such a coexistence of immune activation and immunosuppressive features has been widely reported in tumors with dysfunctional or exhausted immune responses [45,46]. These observations suggest that although PON2-high tumors exhibit increased immune cell infiltration, the antitumor immune activity may be attenuated by compensatory immunosuppressive mechanisms. These correlations were computed across whole tumor tissue. They describe how PON2 and the checkpoint molecules vary together at the tissue level and do not localize their expression to any single cell type, and the functional state of the infiltrating CD8^+^ T cells, including whether they are exhausted, was not assessed. The immune cell populations reported here were estimated from transcriptomic data, and experimental validation will be needed to confirm them. We would also distinguish prognostic association from prediction of treatment response. The survival analyses support a prognostic association between PON2 expression and outcome, but no cohort with patient-level treatment and response data was analysed, so the present study does not establish PON2 as a predictive biomarker for response to any therapy.

This study examines the PON2/CEBPB association in ccRCC, but it has several limitations. First, the gene was identified through bioinformatics screening, relying on transcriptomic data from public databases like TCGA and E-MTAB-1980. This reliance may introduce limitations due to sample heterogeneity and batch effects. Second, while in vitro cell experiments can clarify molecular mechanisms, the tumor microenvironment of ccRCC (such as hypoxia and immune infiltration) is difficult to fully simulate in vitro, necessitating further in vivo experiments to validate the regulatory mechanisms of PON2. Third, the aforementioned single-cell sequencing suggests that PON2 is also localized in endothelial cells in clear cell renal carcinoma, but this article does not further verify the biological function of PON2 in endothelial cells. Fourth, the relationship observed here between PON2, CEBPB and glycolysis is correlative. Further rescue experiments will be required to demonstrate that PON2 regulates CEBPB.

## 5. Conclusions

To summarize, the present findings show marked PON2 upregulation in ccRCC, with elevated expression independently predicting unfavorable patient outcomes. Moreover, our data show an association between high PON2 levels, immunosuppressive features, and an inflamed tumor microenvironment. Furthermore, functional experiments showed that PON2 manipulation was associated with ccRCC phenotypic changes, glycolysis-related metabolic measurements, and CEBPB expression; these changes were restored by wild-type but not S311C PON2.

## Figures and Tables

**Figure 1:**
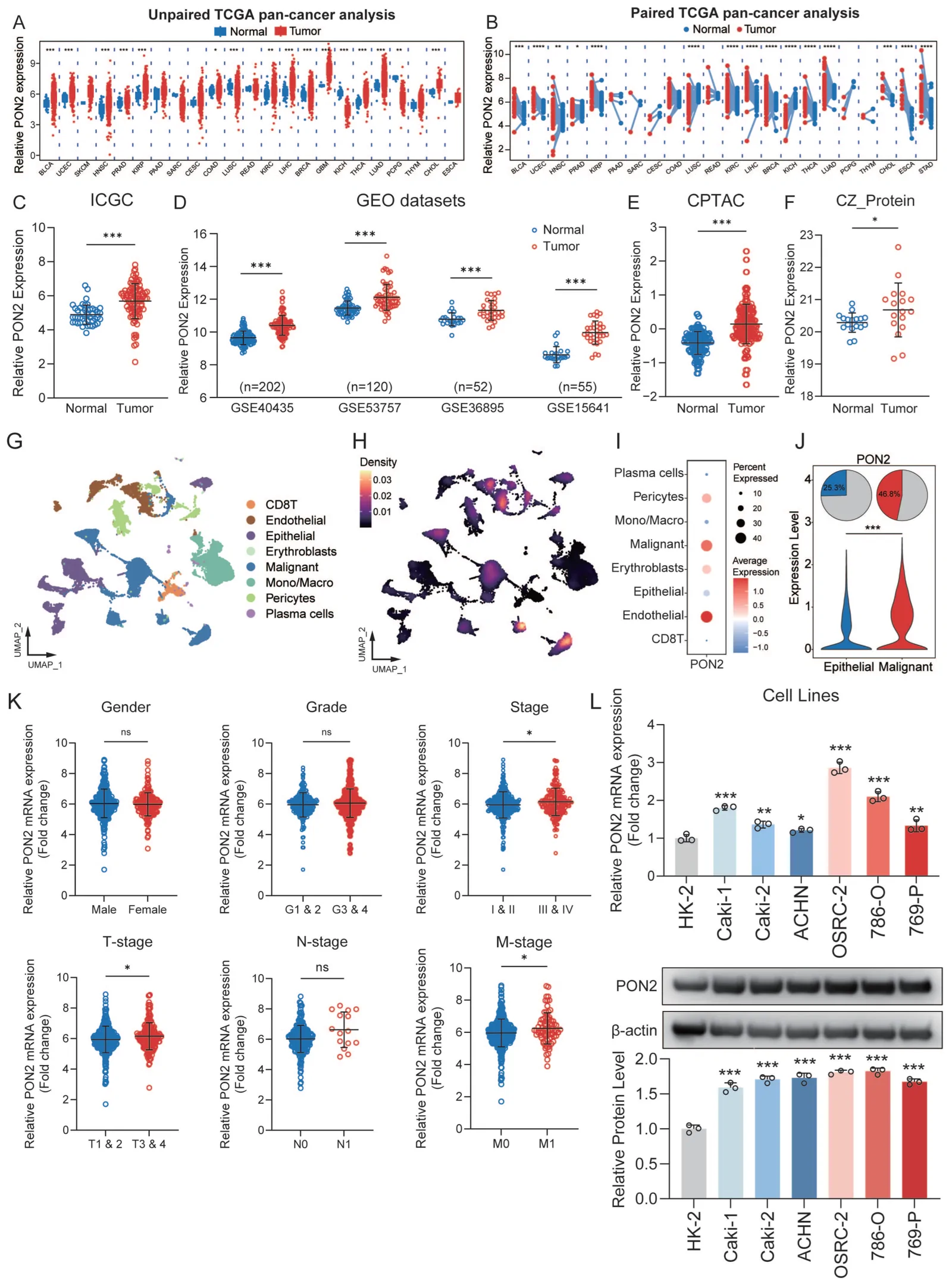
Expression levels of PON2 in various common tumors and renal clear cell carcinoma. (**A**) Expression levels of PON2 in various tumor types from the TCGA database, shown as an unpaired pan-cancer analysis. (**B**) Paired analysis of PON2 in various tumor types from the TCGA database. (**C**–**F**) mRNA and protein expression levels of PON2 in renal clear cell carcinoma tissues and adjacent normal kidney tissues from the ICGC, GEO, CPTAC, and CZ_Protein cohorts. (**G**–**J**) UMAP plot from single-cell sequencing showing the localization of the differentially expressed gene PON2. (**K**) Relationship between PON2 expression levels and clinicopathological features of patients with renal clear cell carcinoma in the TCGA database. (**L**) mRNA and protein expression levels of PON2 in HK2 cells and renal clear cell carcinoma cell lines (ACHN, Caki-1, Caki-2, OSRC-2, 786-O, and 769-P). (Throughout this figure, Normal denotes adjacent normal tissue.) Original western blots are presented in Figure S1. In (**C**–**F**,**K**,**L**), horizontal lines and error bars indicate the mean ± SD. ns, not significant; * p<0.05; ** p<0.01; *** p<0.001; **** p<0.0001.

**Figure 2:**
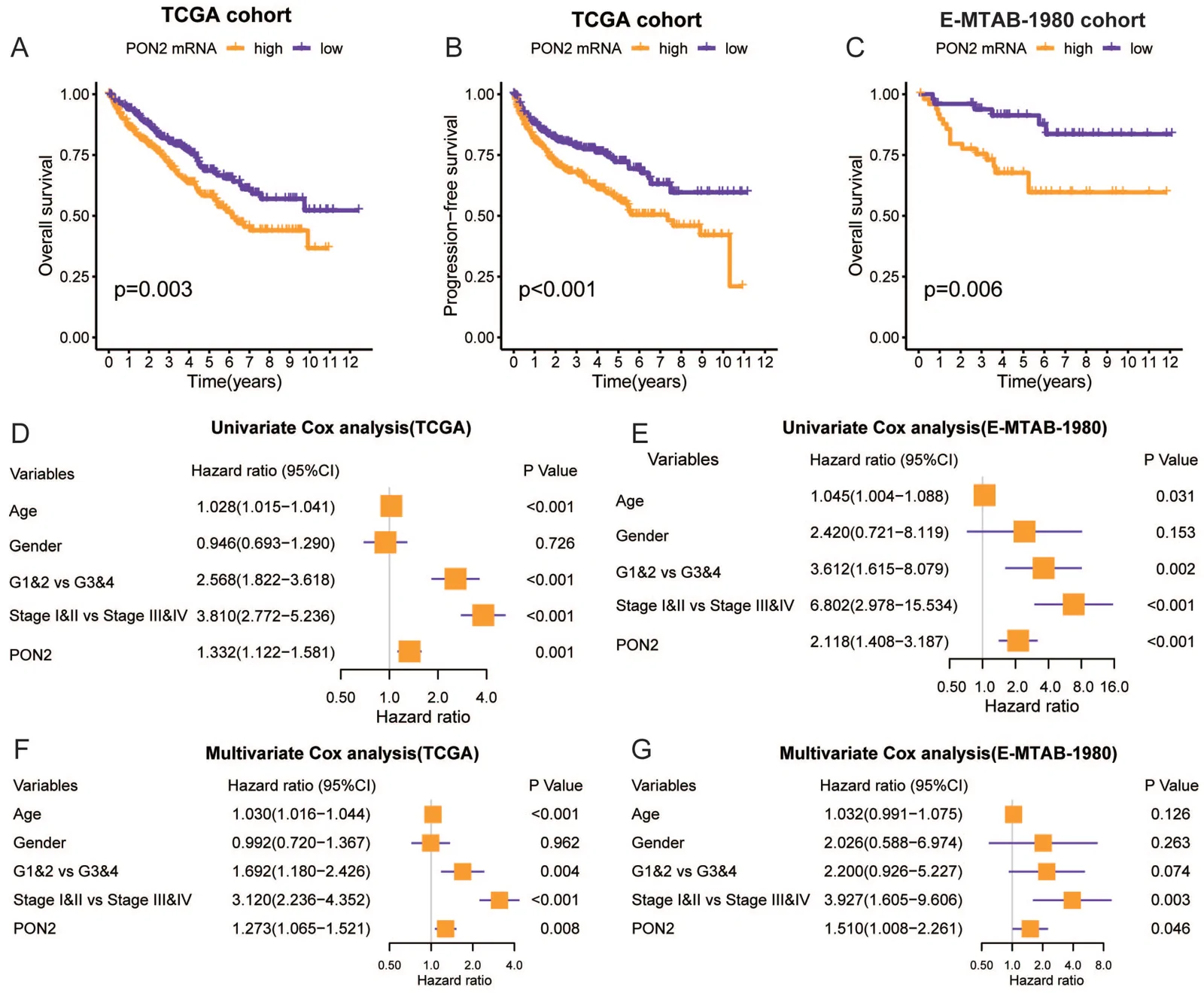
Association of PON2 expression with overall and progression-free survival and its prognostic significance in clear cell renal cell carcinoma (ccRCC). (**A**) Overall survival curve of patients with ccRCC based on PON2 expression levels in the TCGA cohort. (**B**) Progression-free survival curve of patients with ccRCC based on PON2 expression levels in the TCGA cohort. (**C**) Overall survival curve of patients with ccRCC based on PON2 expression levels in the E-MTAB-1980 cohort. (**D**,**E**) Univariate Cox regression analysis of prognostic factors in the TCGA and E-MTAB-1980 cohorts. (**F**,**G**) Multivariate Cox regression analysis in the TCGA and E-MTAB-1980 cohorts identifying independent prognostic factors for ccRCC. In (**D**–**G**), squares indicate hazard ratios, horizontal lines indicate 95% confidence intervals, and the grey vertical line marks a hazard ratio of 1.

**Figure 3:**
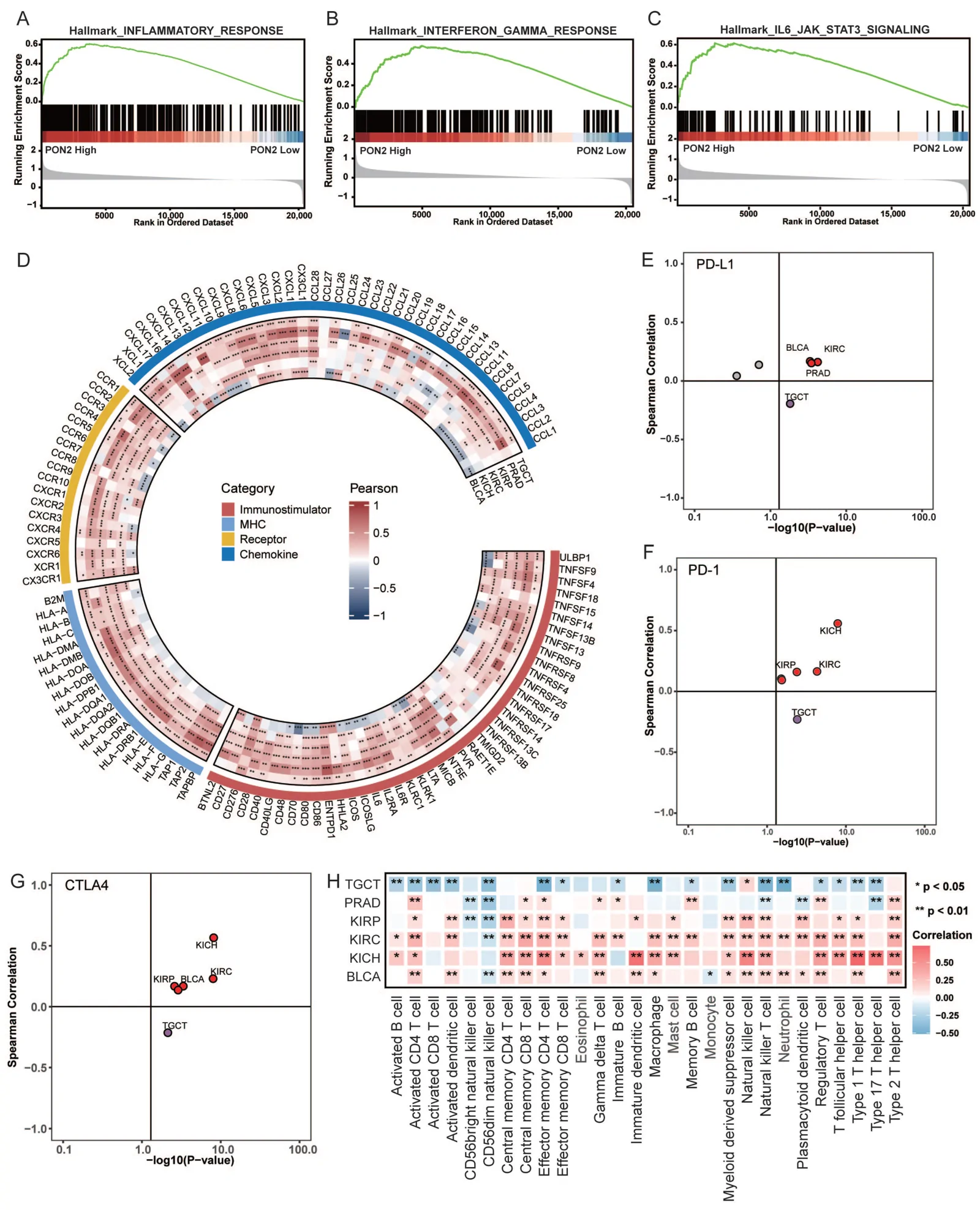
Correlation between PON2 expression and immune landscape in TCGA-KIRC and other urologic tumors. (**A**–**C**) Gene Set Enrichment Analysis (GSEA) reveals that (**A**) inflammatory response, (**B**) interferon-gamma response, and (**C**) IL6-JAK-STAT3 signaling pathways are significantly enriched in the PON2-high expression group in the TCGA-KIRC cohort. (**D**) Circular heatmap illustrating the correlations between PON2 expression and four categories of immune-related genes (immunostimulators, MHC molecules, receptors, and chemokines) across six urologic tumor cohorts (BLCA, KICH, KIRC, KIRP, PRAD, TGCT). Red indicates positive correlation, and blue indicates negative correlation. (**E**–**G**) Analysis of the correlation between PON2 expression and key immune checkpoint molecules: (**E**) PD-L1, (**F**) PD-1, and (**G**) CTLA4. The plots display the Spearman correlation coefficient (*y*-axis) against the significance level (−log_10_ *p*-value, *x*-axis). (**H**) Heatmap depicting the association between PON2 expression and the infiltration levels of 28 immune cell populations (calculated by ssGSEA) across urologic cancers. In (**A**–**C**), the green curve shows the running enrichment score. In (**E**–**G**), red and purple dots indicate cohorts with significant positive and negative correlations, respectively, grey dots indicate cohorts without a significant correlation, and the vertical line marks p=0.05. * p<0.05, ** p<0.01, *** p<0.001.

**Figure 4:**
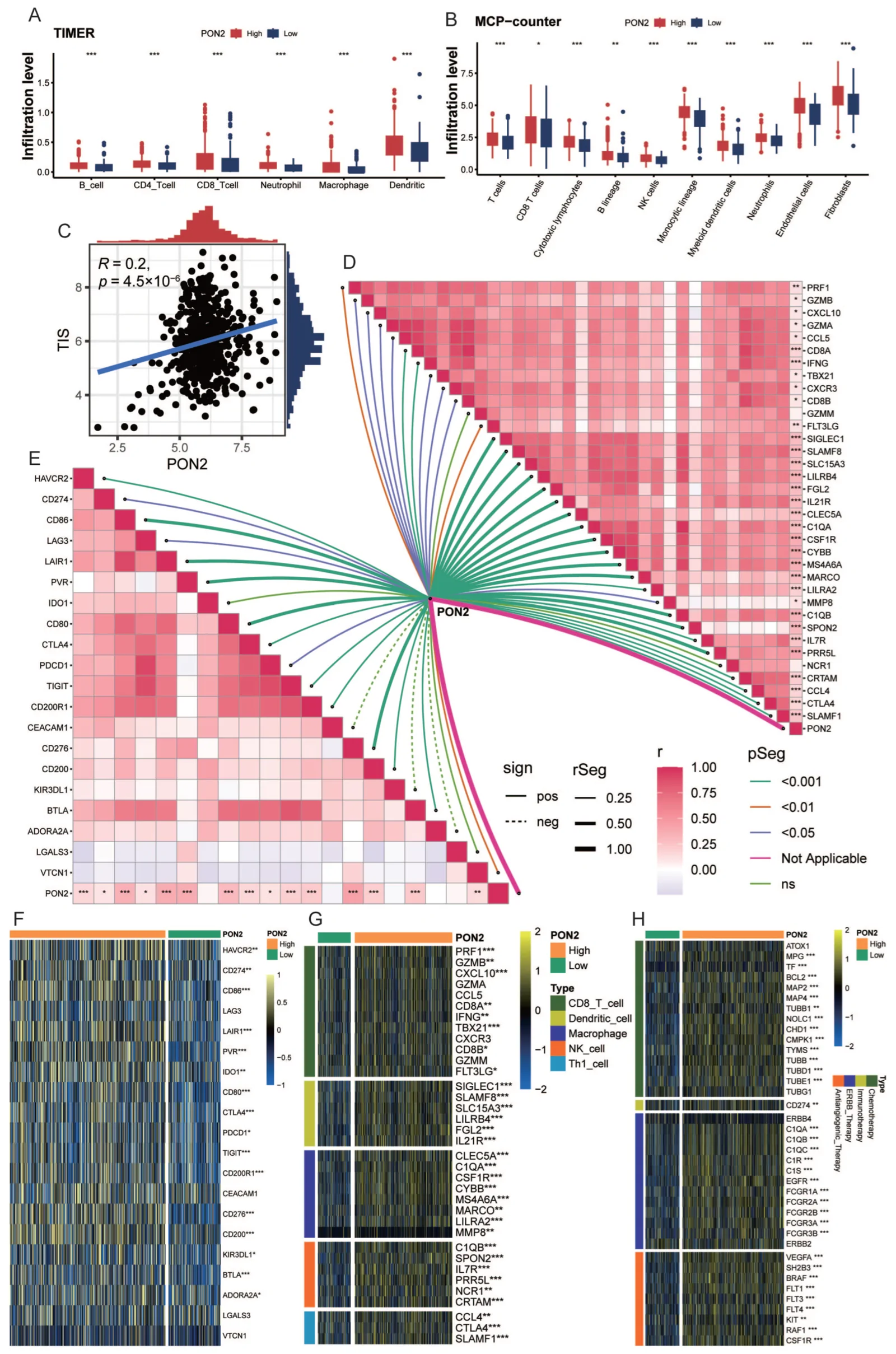
High PON2 expression is associated with higher estimated immune infiltration and an inflamed tumor microenvironment in TCGA-KIRC. (**A**,**B**) Boxplots comparing the infiltration levels of various immune cell populations between PON2-high and PON2-low expression groups in TCGA- KIRC, calculated using the (**A**) TIMER and (**B**) MCP-counter algorithms. (**C**) Scatter plot showing the significant positive correlation between PON2 expression and the T-cell inflamed score (TIS). (**D**) Network diagram and heatmap illustrating the correlations between PON2 expression and immune effector genes related to T-cell cytotoxicity and myeloid cells. The lines in the network diagram indicate the correlation strength and direction. (**E**) Correlation heatmap displaying the relationship between PON2 expression and 21 immune checkpoint molecules. (**F**) Heatmap showing the differential expression levels of immune checkpoint genes between PON2-high and PON2-low groups. (**G**) Heatmap visualizing the expression patterns of immune effector molecules, categorized by cell type (CD8^+^ T cells, dendritic cells, macrophages, NK cells, and Th1 cells), in PON2-high versus PON2-low groups. (**H**) Heatmap depicting the expression trends of therapy-related gene signatures in PON2-high and PON2-low groups. In (**A**,**B**), boxes show the interquartile range, the central line shows the median, whiskers extend to the most extreme values within 1.5 times the interquartile range, and dots show outliers. * p<0.05, ** p<0.01, *** p<0.001.

**Figure 5:**
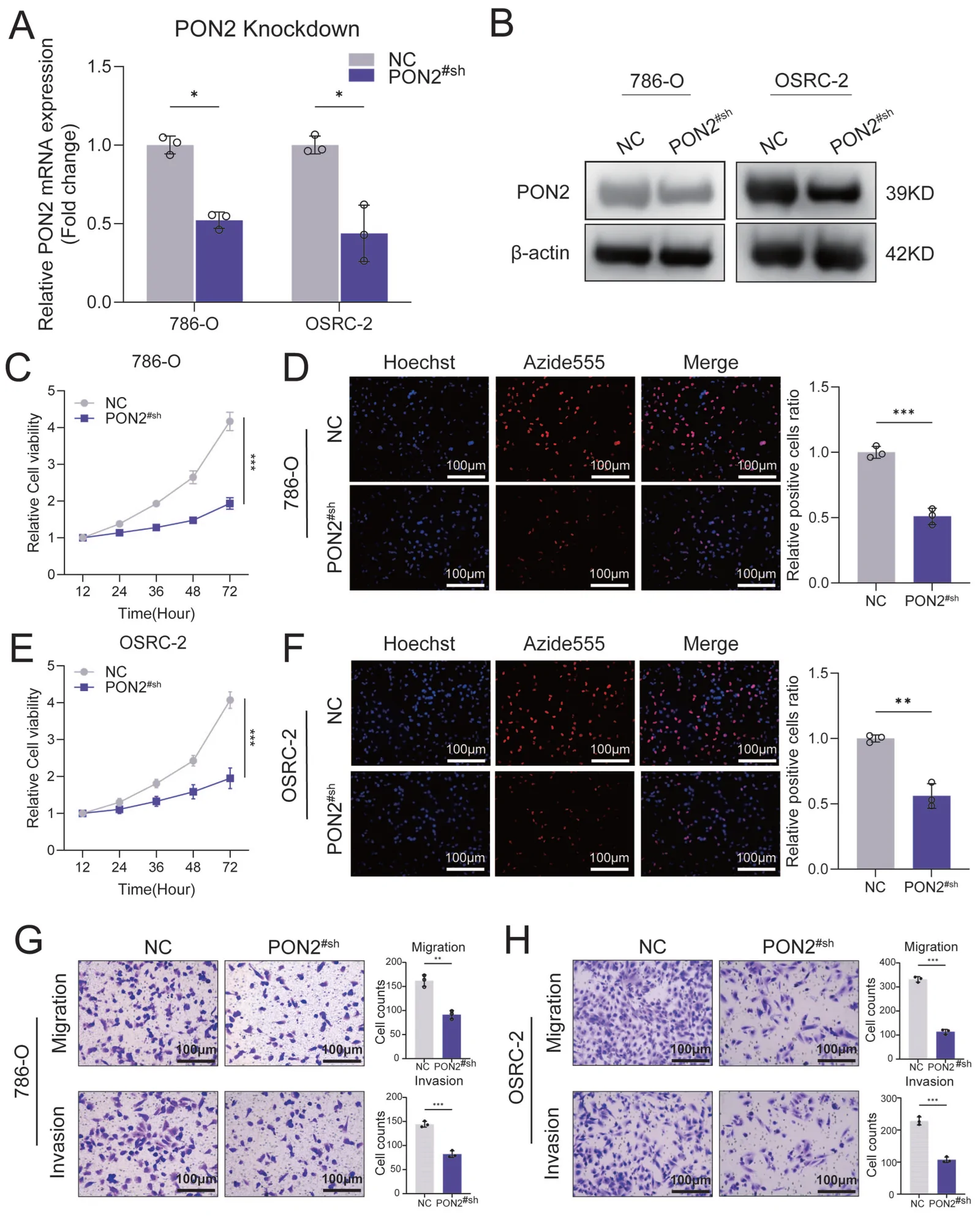
Low expression of PON2 inhibits proliferation, migration, and invasion capabilities of ccRCC cells in vitro. (**A**) mRNA expression levels of NC group and PON2^#sh^ group in 786-O and OSRC-2 cell lines. (**B**) Protein expression levels of NC group and PON2^#sh^ group in 786-O and OSRC-2 cell lines. (**C**) Relative cell viability of NC group and PON2^#sh^ group in the 786-O cell line. (**D**) Comparison of proliferation capabilities between NC group and PON2^#sh^ group in the 786-O cell line; scale bar 100 µm. (**E**) Relative cell viability of NC group and PON2^#sh^ group in the OSRC-2 cell line. (**F**) Comparison of proliferation capabilities between NC group and PON2^#sh^ group in the OSRC-2 cell line; scale bar 100 µm. (**G**) Comparison of migration and invasion capabilities between NC group and PON2^#sh^ group in the 786-O cell line; scale bar 100 µm. (**H**) Comparison of migration and invasion capabilities between NC group and PON2^#sh^ group in the OSRC-2 cell line; scale bar 100 µm. Original western blots are presented in Figure S2. Data are presented as the mean ± SD of three independent experiments. * p<0.05, ** p<0.01, *** p<0.001 versus NC.

**Figure 6:**
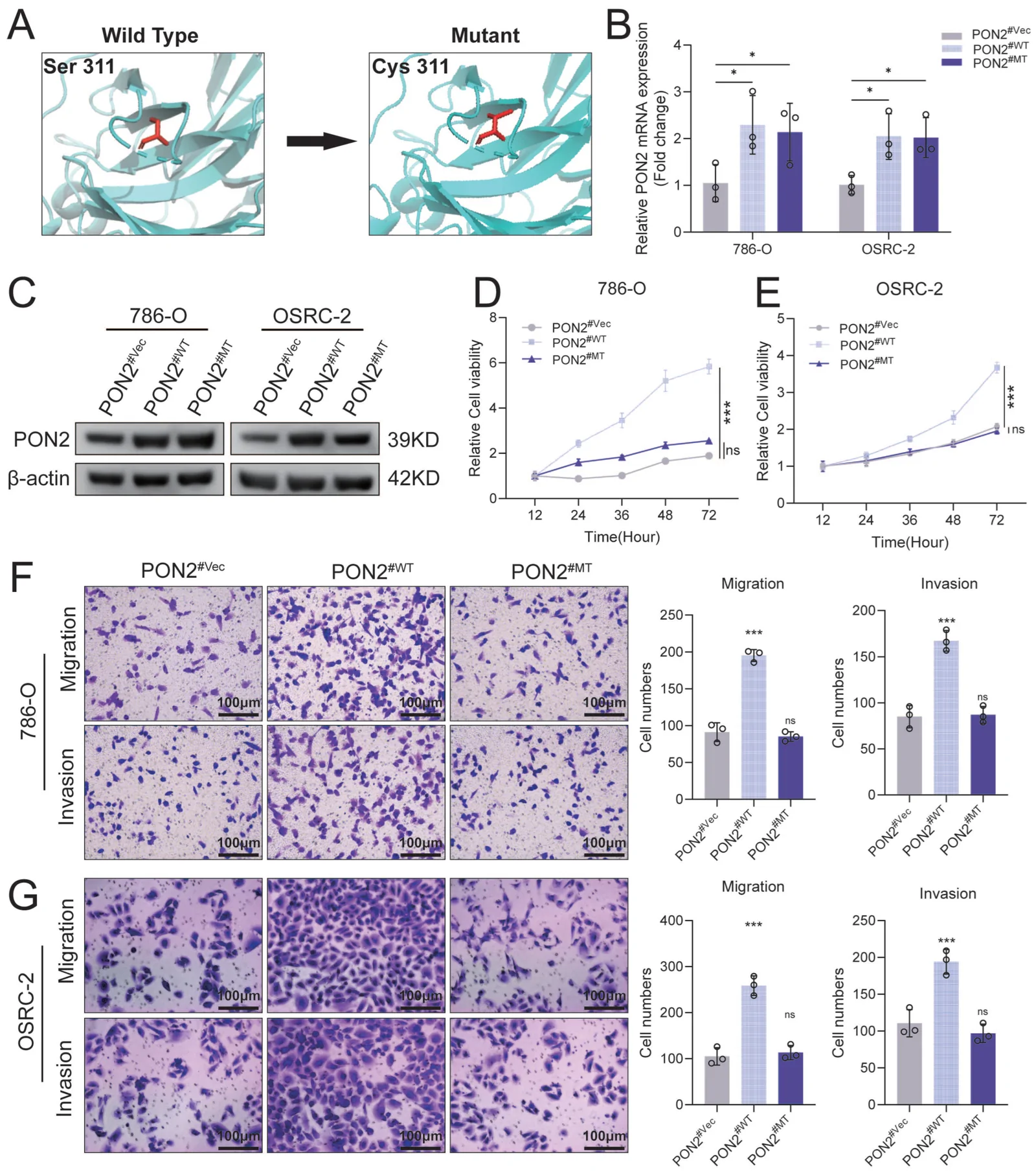
Reconstitution with wild-type but not S311C PON2 restores proliferation, migration and invasion capabilities of ccRCC cells in vitro. (**A**) Schematic diagram of mutation sites for the S311C mutant (PON2^#MT^). (**B**) mRNA expression levels of PON2^#Vec^ group, PON2^#WT^ group, and PON2^#MT^ group in 786-O and OSRC-2 cell lines. (**C**) Protein expression levels of PON2^#Vec^ group, PON2^#WT^ group, and PON2^#MT^ group in 786-O and OSRC-2 cell lines. (**D**) Relative cell viability of PON2^#Vec^ group, PON2^#WT^ group, and PON2^#MT^ group in the 786-O cell line. (**E**) Relative cell viability of PON2^#Vec^ group, PON2^#WT^ group, and PON2^#MT^ group in the OSRC-2 cell line. (**F**) Comparison of migration and invasion capabilities among PON2^#Vec^ group, PON2^#WT^ group, and PON2^#MT^ group in the 786-O cell line; scale bar 100 µm. (**G**) Comparison of migration and invasion capabilities among PON2^#Vec^ group, PON2^#WT^ group, and PON2^#MT^ group in the OSRC-2 cell line; scale bar 100 µm. Original western blots are presented in Figure S3. In (**A**), the PON2 backbone is shown in light green and residue 311 in red. Data are presented as the mean ± SD of three independent experiments. * p<0.05, *** p<0.001 versus PON2^#Vec^; ns, not significant.

**Figure 7:**
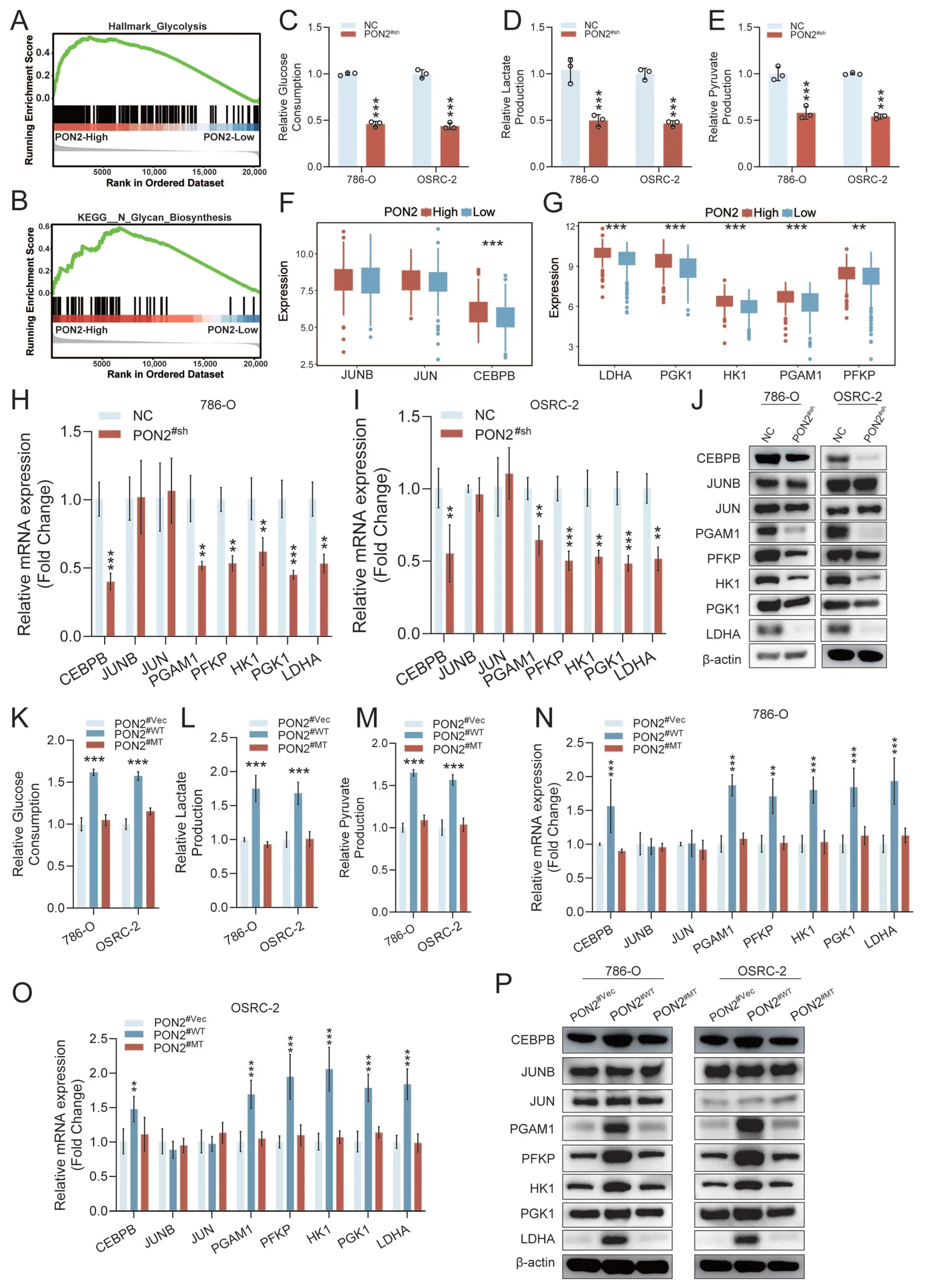
PON2 is associated with ccRCC progression and CEBPB-related glycolysis. (**A**,**B**) GSEA functional enrichment analysis. (**C**–**E**) Measurement of glycolysis-related metabolic parameters in NC group and PON2^#sh^ group in 786-O and OSRC-2 cell lines. (**F**,**G**) Analysis of expression differences between PON2 and key enzymes regulating glycolysis (PGAM1, PFKP, HK1, PGK1, LDHA) and their upstream transcription factors (CEBPB, JUNB, JUN). (**H**) mRNA expression levels of key enzymes regulating glycolysis and their upstream transcription factors in NC group and PON2^#sh^ group in the 786-O cell line. (**I**) mRNA expression levels of key enzymes regulating glycolysis and their upstream transcription factors in NC group and PON2^#sh^ group in the OSRC-2 cell line. (**J**) Protein expression levels of key enzymes regulating glycolysis and their upstream transcription factors in NC group and PON2^#sh^ group in 786-O and OSRC-2 cells. (**K**–**M**) Measurement of glycolysis-related metabolic parameters in PON2^#Vec^ group, PON2^#WT^ group, and PON2^#MT^ group in 786-O and OSRC-2 cells. (**N**) mRNA expression levels of key enzymes regulating glycolysis and their upstream transcription factors in PON2^#Vec^ group, PON2^#WT^ group, and PON2^#MT^ group in the 786-O cell line. (**O**) mRNA expression levels of key enzymes regulating glycolysis and their upstream transcription factors in PON2^#Vec^ group, PON2^#WT^ group, and PON2^#MT^ group in the OSRC-2 cell line. (**P**) Protein expression levels of key enzymes regulating glycolysis and their upstream transcription factors in PON2^#Vec^ group, PON2^#WT^ group, and PON2^#MT^ group in 786-O and OSRC-2 cells. Original western blots are presented in Figures S4 and S5. Boxplots in (**F**,**G**) are drawn as described for Figure 4. Data in (**C**–**E**,**H**,**I**,**K**–**O**) are presented as the mean ± SD of three independent experiments. ** p<0.01, *** p<0.001 versus the PON2-low group (**F**,**G**), NC (**C**–**E**,**H**,**I**) or PON2^#Vec^ (**K**–**O**).

## Data Availability

The datasets presented in this study can be found in online repositories. The pan-cancer RNA-seq and clinical data were obtained from the UCSC Xena website (https://xena.ucsc.edu/, accessed on 5 January 2025). Proteomic data for ccRCC were retrieved from the CPTAC database (https://cptac-data-portal.georgetown.edu/, accessed on 15 January 2022). Validation was performed using data from the ICGC database (https://dcc.icgc.org/, accessed on 7 March 2024) and the GEO database under accession numbers GSE40435, GSE53757, GSE36895, and GSE15641. Additionally, single-cell RNA-sequencing data (GSE159115) were accessed via the TISCH database (http://tisch.comp-genomics.org/, accessed on 5 January 2025). The independent E-MTAB-1980 ccRCC expression and survival cohort was obtained from ArrayExpress/EMBL-EBI (https://www.ebi.ac.uk/biostudies/arrayexpress, accessed on 5 January 2025). The CZ_Protein data produced herein will be made available by the corresponding author upon reasonable request.

## Supplementary Materials

The following supporting information can be downloaded at https://www.mdpi.com/article/10.3390/cancers18193086/s1, Table S1: Primers sequences used in this work; Figure S1: Original images of western blots for Figure 1L, bottom panel; Figure S2: Original images of western blots for Figure 5B; Figure S3: Original images of western blots for Figure 6C; Figure S4: Original images of western blots for Figure 7J; Figure S5: Original images of western blots for Figure 7P.

## Abbreviations

The following abbreviations are used in this manuscript:

ccRCC	Clear cell renal cell carcinoma
CPTAC	Clinical Proteomic Tumor Analysis Consortium
EMT	Epithelial–mesenchymal transition
GEO	Gene Expression Omnibus
GSEA	Gene Set Enrichment Analysis
ICB	Immune checkpoint blockade
ICGC	International Cancer Genome Consortium
KIRC	Kidney renal clear cell carcinoma (TCGA cohort)
MDSC	Myeloid-derived suppressor cell
OS	Overall survival
PFS	Progression-free survival
PON2	Paraoxonase 2
RCC	Renal cell carcinoma
ssGSEA	Single-sample gene set enrichment analysis
TCGA	The Cancer Genome Atlas
TIS	T-cell inflamed score
TISCH	Tumor Immune Single-cell Hub
TME	Tumor microenvironment
TPM	Transcripts per kilobase million
Treg	Regulatory T cell
UMAP	Uniform Manifold Approximation and Projection
