# Supplementary material for: Association of PON2 and Its S311C Substitution with Tumor Progression and Its Significance as an Immune-Associated Prognostic Indicator in Clear Cell Renal Cell Carcinoma
Source: Cancers (Basel). 2026 Sep 23;18(19):3086. doi: 10.3390/cancers18193086 (PMC13664073; doi:10.3390/cancers18193086)
Supplement: Supplementary file 1 [file cancers-18-03086-s001.zip › Figures S1-S5.pdf]

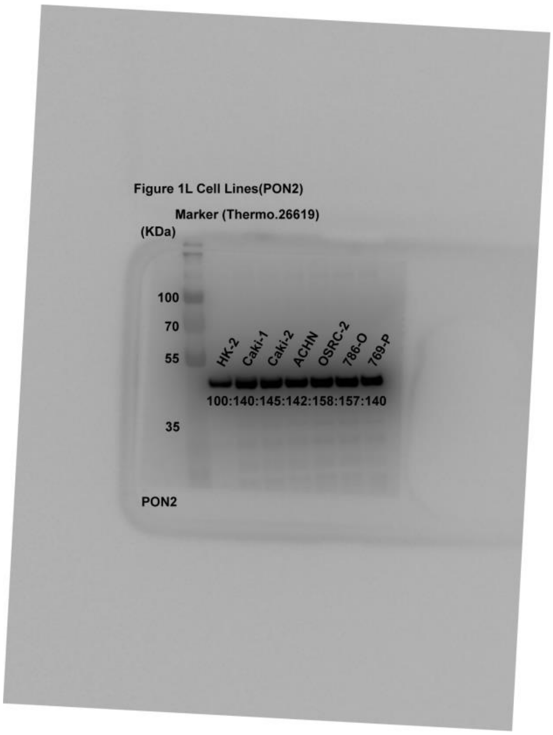

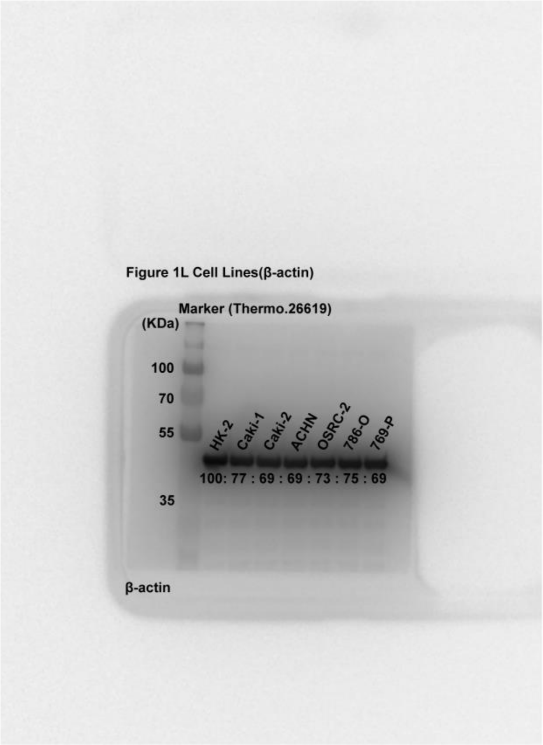

Figure S1 Original images of western blots for Figure 1L, bottom panel

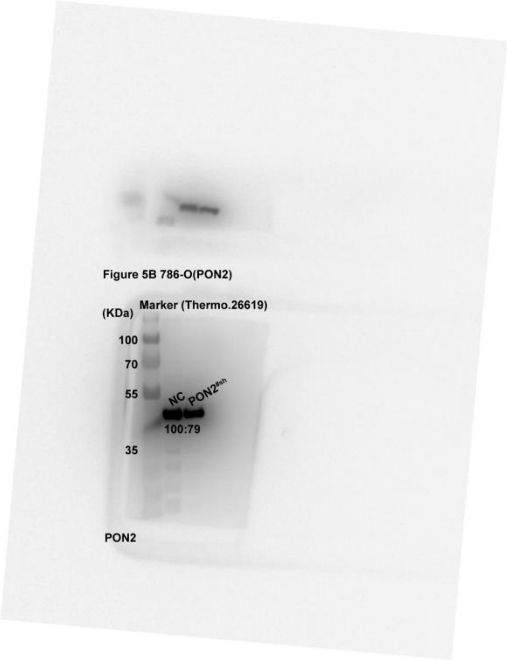

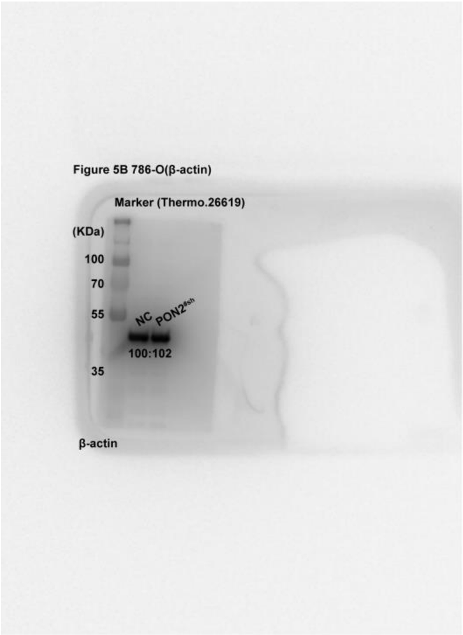

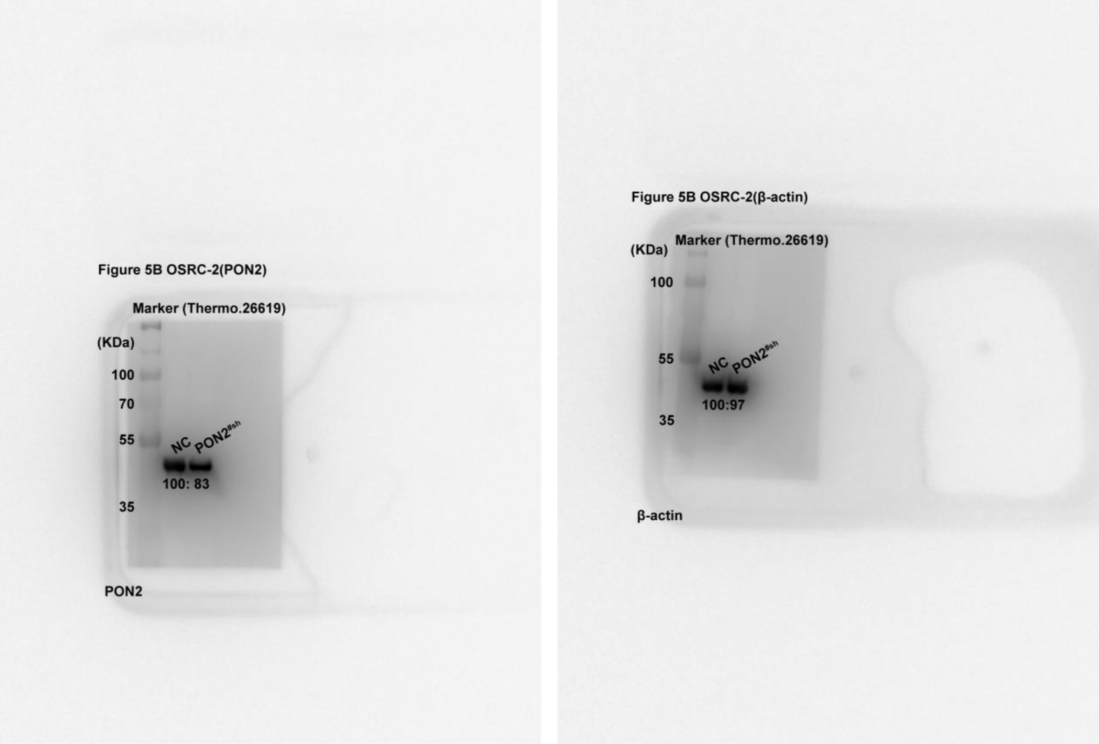

Figure S2 Original images of western blots for Figure 5B

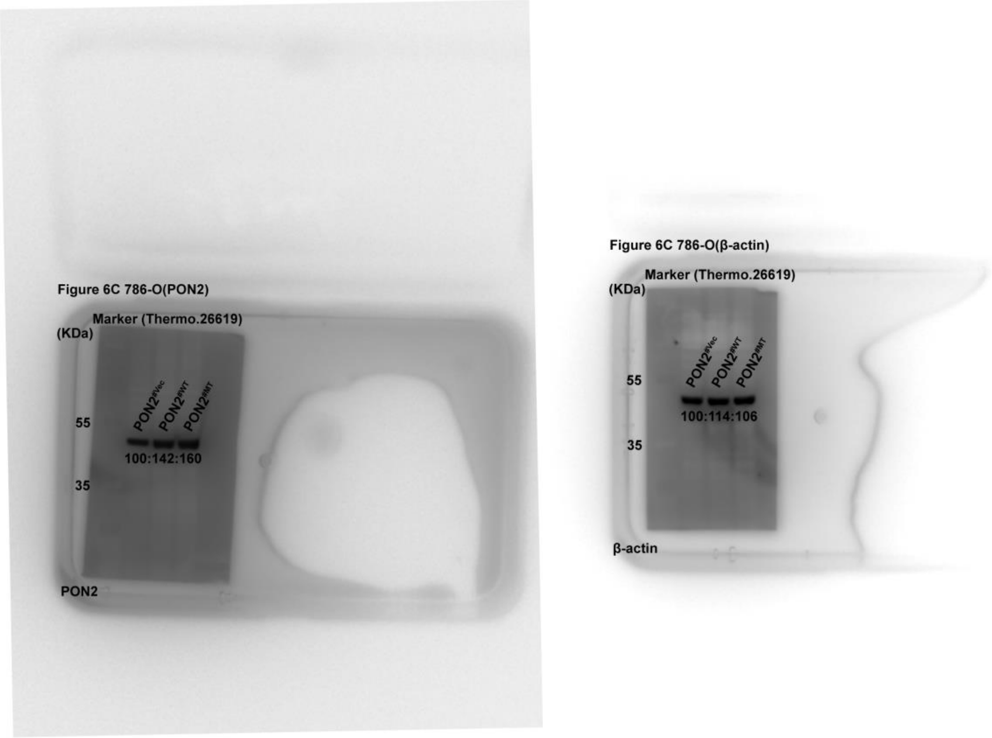

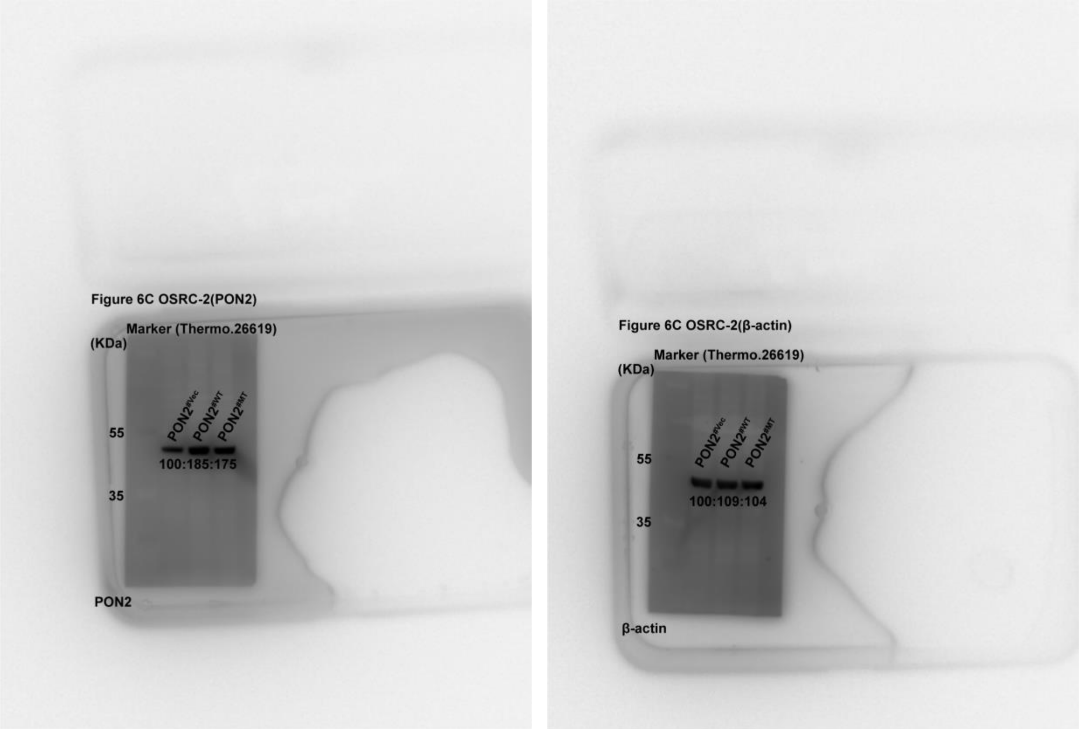

Figure S3 Original images of western blots for Figure 6C

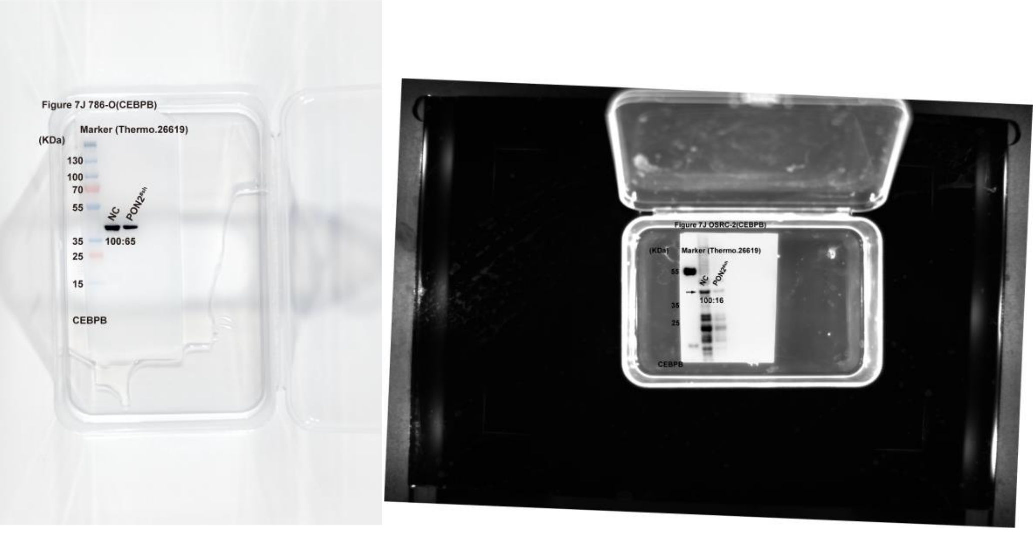

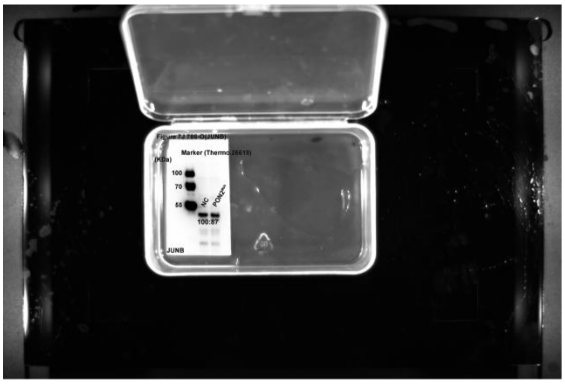
Figure 7J 786-O(JUNB)
Marker (Thermo.26619)
(KDa)
100
70
55
NC
PON2
100:87
JUNB

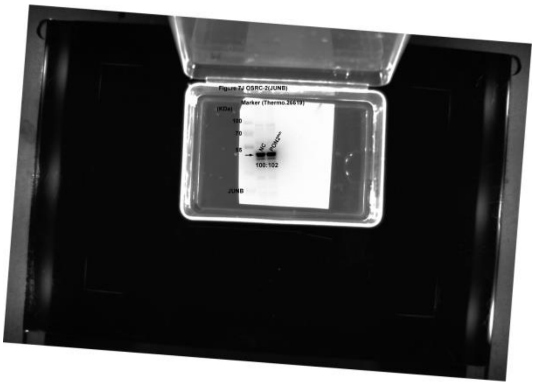
Figure 7J OSRC-2(JUNB)
Marker (Thermo.26619)
(KDa)
100
70
55
NC
PON2
100:102
JUNB

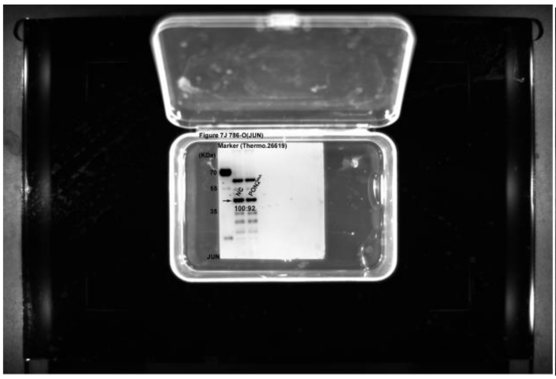
Figure 7J 786-O(JUN)
Marker (Thermo.26619)
(KDa)
70
55
35
NC
PON2
100:92
JUN

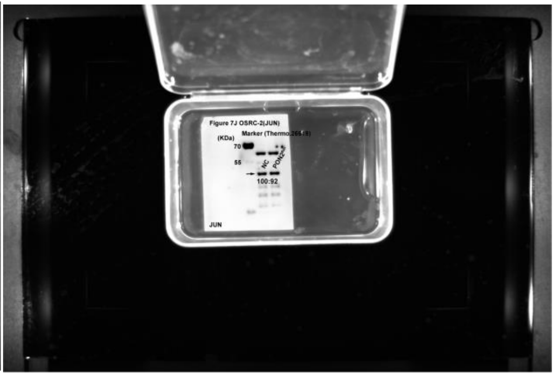
Figure 7J OSRC-2(JUN)
Marker (Thermo.26619)
(KDa)
70
55
NC
PON2
100:92
JUN

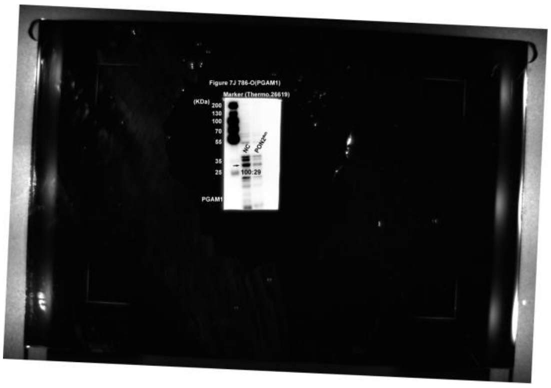
(KDa)
200
130
100
70
55
35
25
NC
PON2
100:29
PGAM1

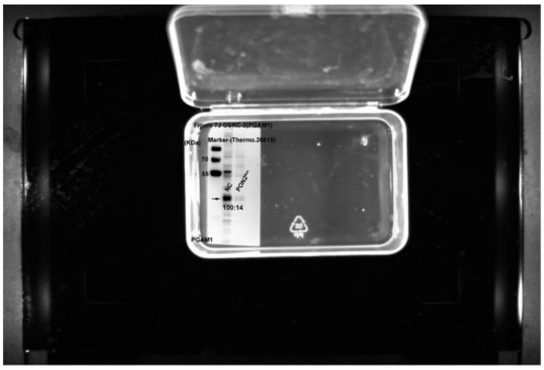
(KDa)
70
55
NC
PON2
100:14
PGAM1

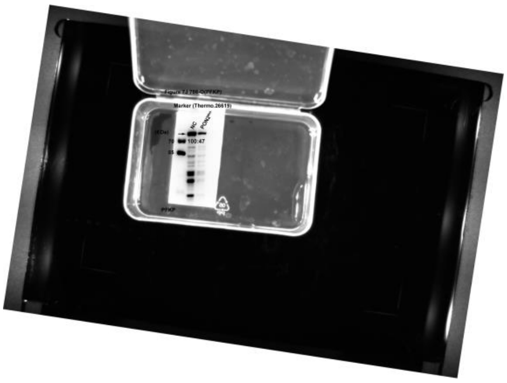
NC
PON2
100:47

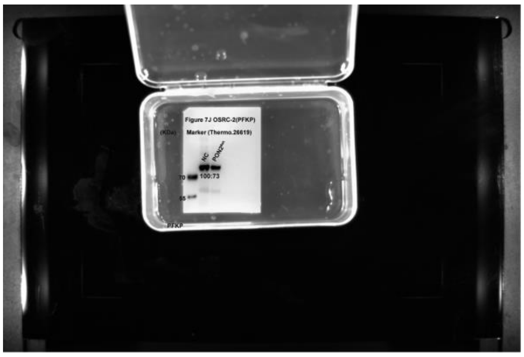
Figure 7J OSRC-2(PFKP)
(KDa)
Marker (Thermo.26619)
NC
PON2
100:73
70
55
PFKP

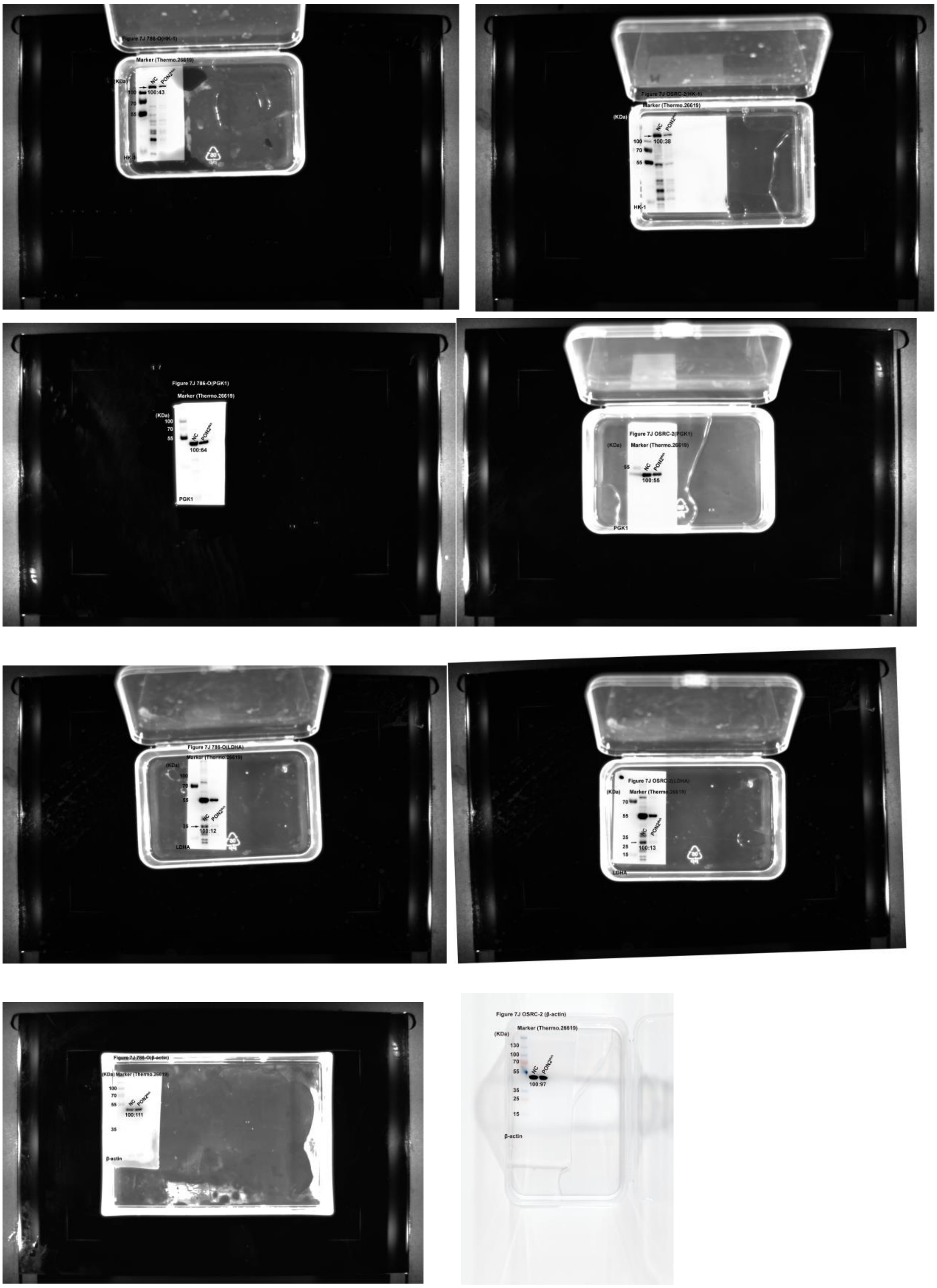

Figure S4 Original images of western blots for Figure 7J

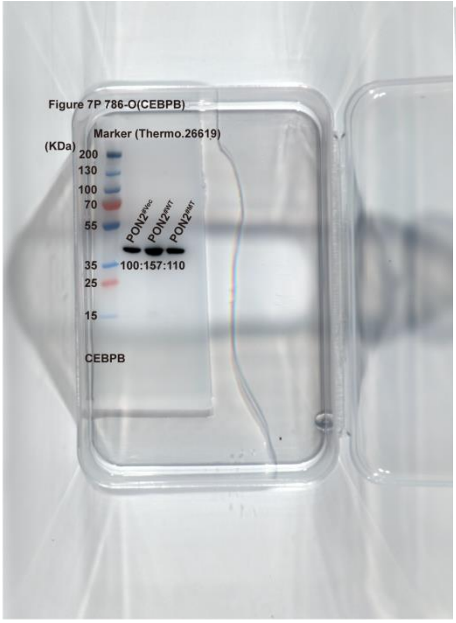
Figure 7P 786-O(CEBPB)
Marker (Thermo.26619)
(KDa)
200
130
100
70
55
35
25
15
$PON2^{Vec}$
$PON2^{WT}$
$PON2^{MT}$
100:157:110
CEBPB

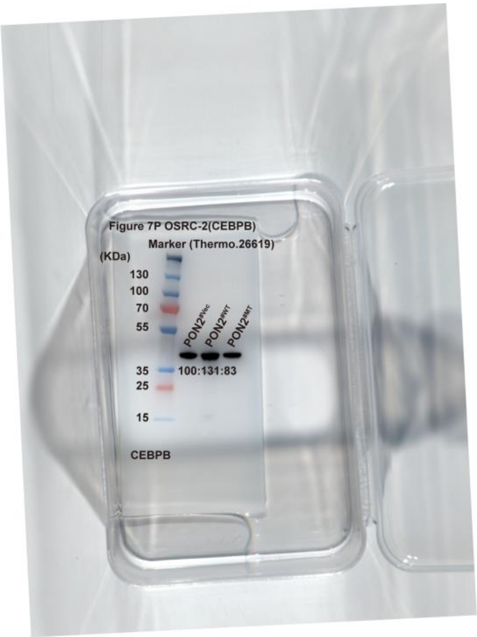
Figure 7P OSRC-2(CEBPB)
Marker (Thermo.26619)
(KDa)
130
100
70
55
35
25
15
$PON2^{Vec}$
$PON2^{WT}$
$PON2^{MT}$
100:131:83
CEBPB

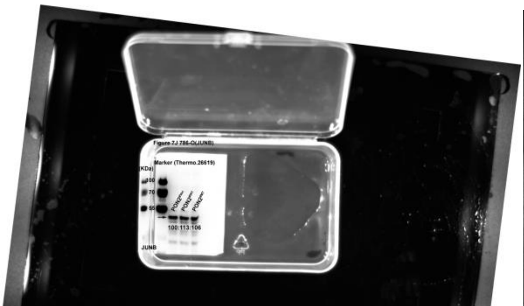
Figure 7J 786-O(JUNB)
Marker (Thermo.26619)
(KDa)
100:113:106
JUNB

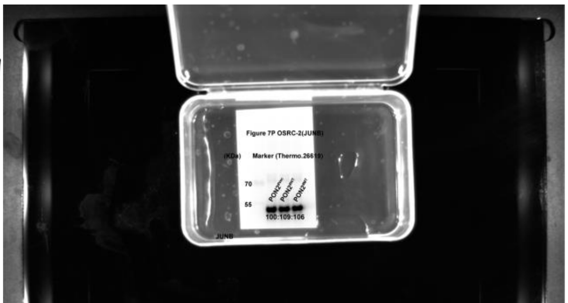
Figure 7P OSRC-2(JUNB)
(KDa)
Marker (Thermo.26619)
70
55
100:109:106
JUNB

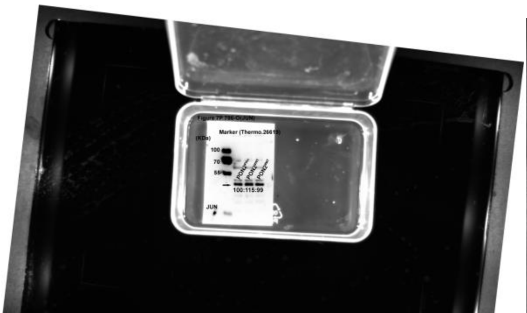
Figure 7P 786-O(JUN)
(KDa)
100
70
55
100:115:99
JUN

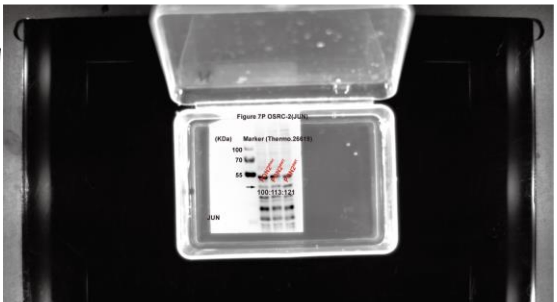
Figure 7P OSRC-2(JUN)
(KDa)
Marker (Thermo.26619)
100
70
55
100:113:121
JUN

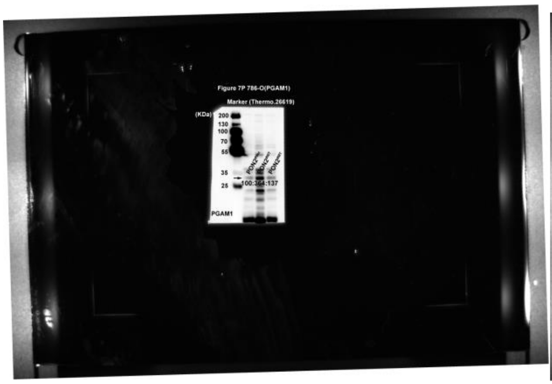
Figure 7P 786-O(PGAM1)
Marker (Thermo.26619)
(KDa)
200
130
100
70
55
35
25
PGAM1

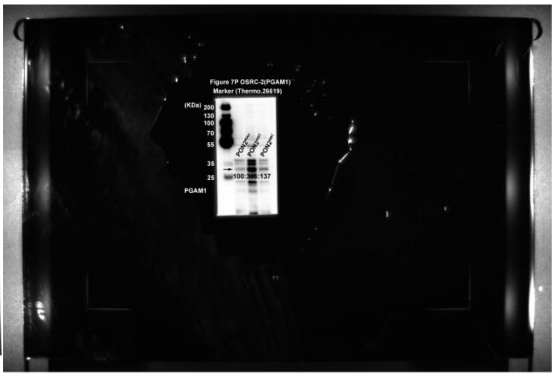
Figure 7P OSRC-2(PGAM1)
Marker (Thermo.26619)
(KDa)
200
130
100
70
55
35
25
PGAM1

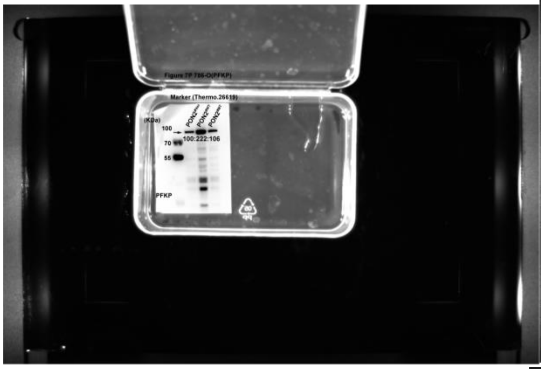
Figure 7P 786-O(PFKP)
Marker (Thermo.26619)
(KDa)
100
70
55
100:222:106
PFKP

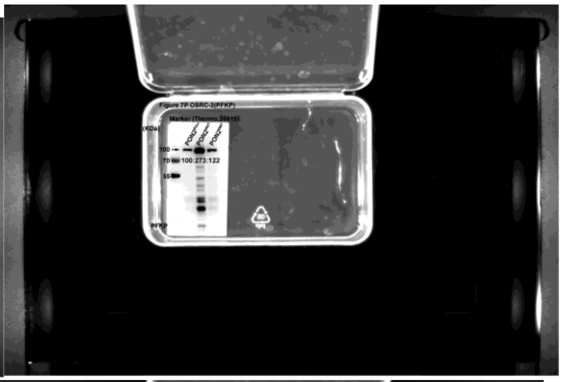
Figure 7P OSRC-2(PFKP)
(KDa)
100
70
55
100:273:122
PFKP

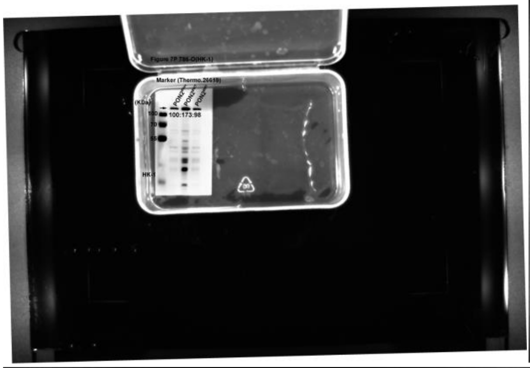
Marker (Thermo.26619)
(KDa)
100:173:98

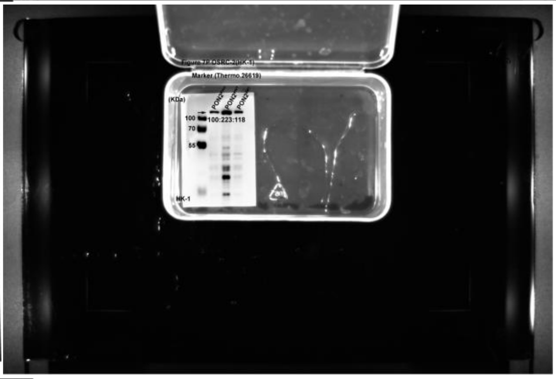
Marker (Thermo.26619)
(KDa)
100
70
55
100:223:118
HK-1

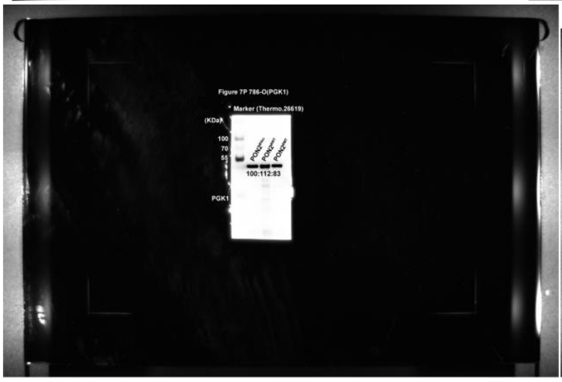
Figure 7P 786-O(PGK1)
Marker (Thermo.26619)
(KDa)
100
70
55
100:112:83
PGK1

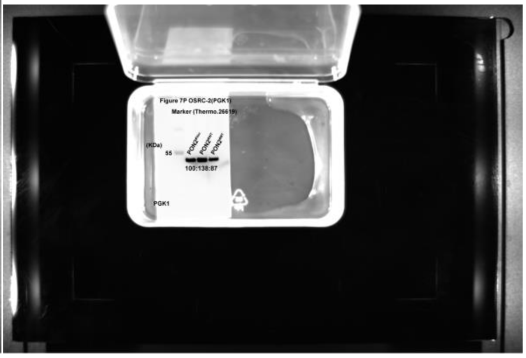
Figure 7P OSRC-2(PGK1)
Marker (Thermo.26619)
(KDa)
55
100:138:87
PGK1

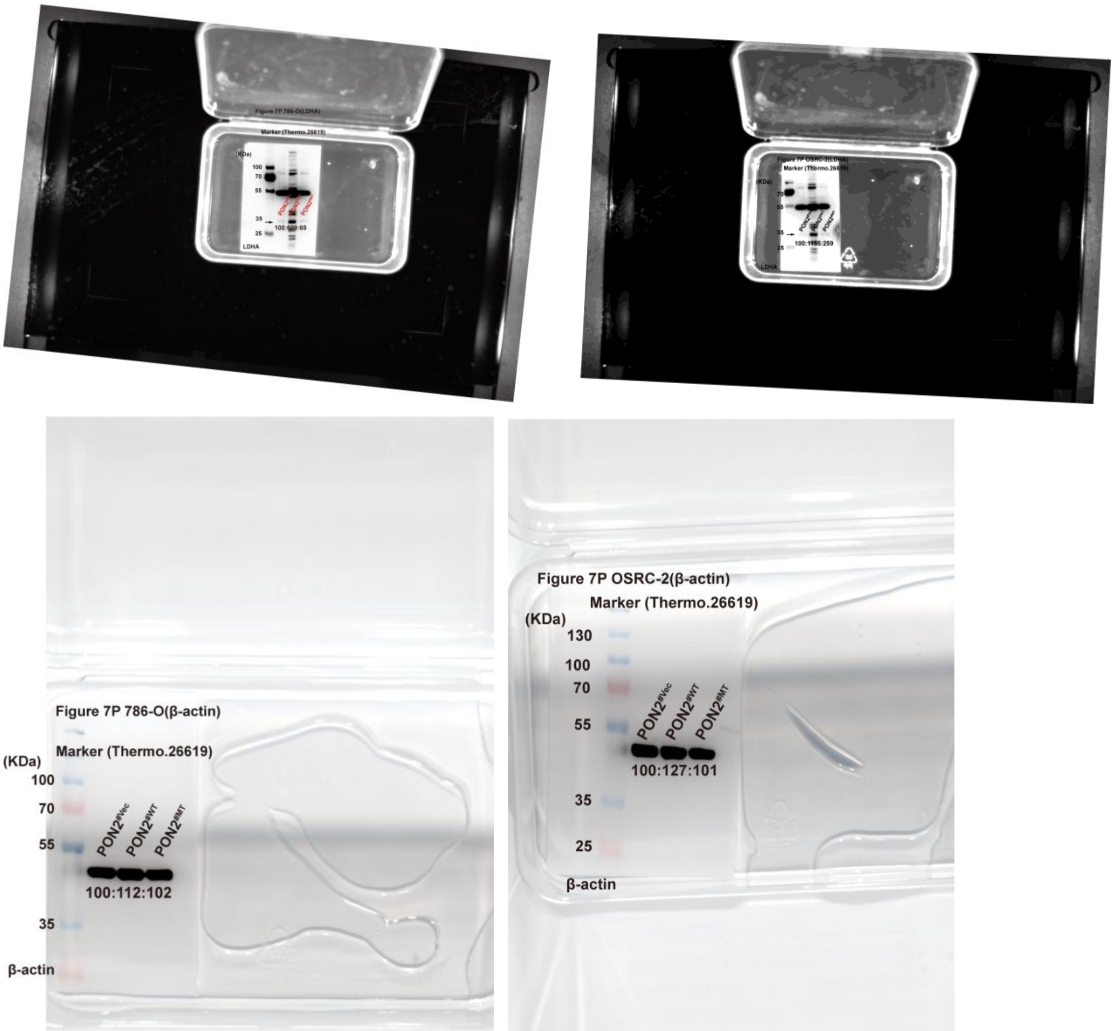

Figure S5 Original images of western blots for Figure 7P
